# Last Decade of Unconventional Methodologies for the Synthesis of Substituted Benzofurans

**DOI:** 10.3390/molecules25102327

**Published:** 2020-05-16

**Authors:** Lucia Chiummiento, Rosarita D’Orsi, Maria Funicello, Paolo Lupattelli

**Affiliations:** Department of Science, Via dell’Ateneo Lucano, 10, 85100 Potenza, Italy; rosarita.dorsi@gmail.com (R.D.); maria.funicello@unibas.it (M.F.); paolo.lupattelli@unibas.it (P.L.)

**Keywords:** synthesis of benzofurans, intra-molecular approach, inter-molecular approach

## Abstract

This review describes the progress of the last decade on the synthesis of substituted benzofurans, which are useful scaffolds for the synthesis of numerous natural products and pharmaceuticals. In particular, new intramolecular and intermolecular C–C and/or C–O bond-forming processes, with transition-metal catalysis or metal-free are summarized. (1) Introduction. (2) Ring generation via intramolecular cyclization. (2.1) C7a–O bond formation: (route a). (2.2) O–C2 bond formation: (route b). (2.3) C2–C3 bond formation: (route c). (2.4) C3–C3a bond formation: (route d). (3) Ring generation via intermolecular cyclization. (3.1) C7a-O and C3–C3a bond formation (route a + d). (3.2) O–C2 and C2–C3 bond formation: (route b + c). (3.3) O–C2 and C3–C3a bond formation: (route b + d). (4) Benzannulation. (5) Conclusion.

## 1. Introduction

**Figure 1:**
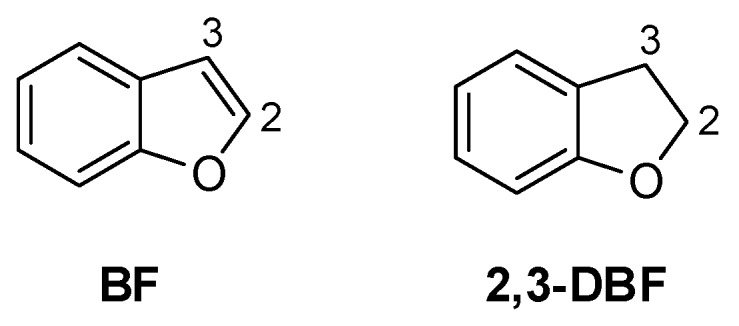
Structures of benzofuran (**BF**) and 2,3-dihydrobenzofuran (**2,3-DBF**).

Benzofuran (**BF**) and 2,3-dihydrobenzofuran (**2,3-DBF**) are key structural units in a variety of biologically active natural products (Figure 1) and represent the core structures of many approved drugs, as well as lead-design developments from natural products [1,2,3,4].

**Figure 2:**
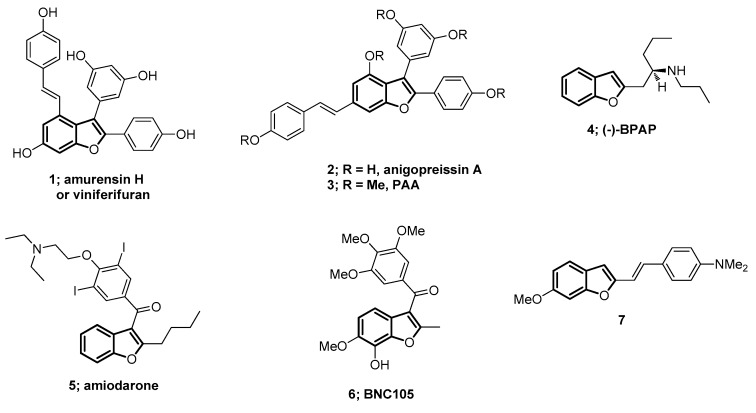
Representative pharmacologically active BF derivatives.

BF is a completely aromatic flat structure, while 2,3-DBF bears two prochiral sp^3^ carbons on the heterocycle, placing the substituents out of the benzofuryl plane. Naturally occurring compounds bearing BF and their derivatives show a broad range of pharmacological activities. Among them, amurensin H (or viniferifuran) **1** (Figure 2) displayed anti-inflammatory effect on an asthma-like reaction induced in mice [5], while Anigopreissin A **2** showed low antimicrobial activity against *Staphylococcus. aureus* and *S. pyogene* [6] and was also discovered as an inhibitor of HIV-1 reverse transcriptase (IC50 = 8 mM), including two mutant enzymes resistant to the clinical drug nevirapine [7].

Permethylated anigopreissin A (PAA) **3** showed inhibitory activity for human hepatoma cell proliferation [8,9], while different benzofuran derivatives have shown pharmacological properties such as anticancer [10,11], antiviral [12,13], anti-Alzheimer′s disease [14,15], together with antiparasitic [16], antitubercular [17], and antibacterial [18,19] activities. Prescribed agents featuring the benzofuran scaffold include the antidepressant (−)-BPAP **4**, the antiarrythmic amiodarone **5** [20], the clinical candidate drug for renal and ovarian cancers BNC105 **6** [21], and the inhibitor of Aβ fibril formation **7** [2]. Such a variety of biological and pharmacological activities make BF an important pharmacophore for the development of new drugs.

Thus, synthetic access to benzofurans is of considerable interest, and numerous approaches to this scaffold have been disclosed in the literature. Herein, we deal with the most recent literature, which is not included in the appeared reviews of the last decade (2009–2020) [22].

The review of De Luca et al. [23] dealt with the synthesis of 2-substituted-benzofurans up to 2009, while the one of Abu-Hashem et al. [24] was an overview about the different approaches to benzofurans. Very recently, from the same authors, a chapter in Advances in Heterocyclic Chemistry [25] and three reviews have been published in which the full perspective of reactivity of benzofurans [26], advances in the synthesis of biologically potent compounds bearing at least one benzofuran moiety in their structures [27] and the recent reports on the total synthesis of natural products containing at least one benzofuran moiety in their complex structures [28] have been discussed. A review on synthetic routes for synthesis of benzofuran-based compounds appeared in 2017 [29] and at the least one summarizes the recent studies on the various aspects of benzofurans derivatives, including their natural sources, biological activities and drug prospects, and chemical synthesis, as well as the relationship between the bioactivities and structures [30].

In this plethora of methodologies, different classifications were used, subdividing the data into synthesis of 2- or 3-substituted or 2,3-disubstituted benzofurans [31,32], or into transition-metal-catalyzed [33,34,35,36,37] vs. metal-free approaches, or pointing out the most recent applications of metal catalyzed C–H insertion [38]. We chose to compare the intra-molecular and inter-molecular methodologies used to build selected bonds.

**Scheme 1:**
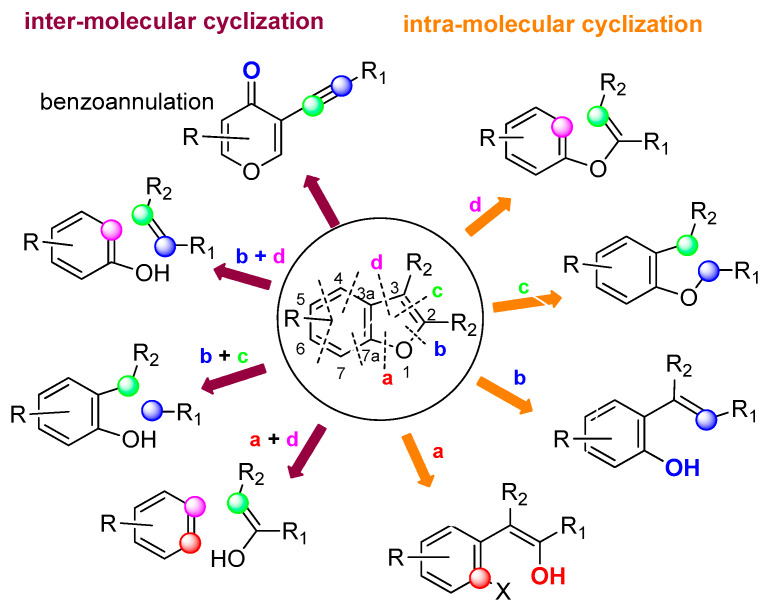
Intra-molecular and inter-molecular retrosynthetic approaches to benzofuran scaffold.

In this review, the synthetic approaches are classified according to the method by which the core BF structure is constructed. We divided the methods into intra-molecular and inter-molecular approaches. Both are classified according to which bond is formed in the key reaction (Scheme 1).

Intra-molecular approaches are the most dated and commonly used, so only newly developed catalytic systems and reaction conditions are introduced herein. The real novelty of this decade is represented by the advent of the inter-molecular strategies often employing transition metal as catalysts (Rh, Fe, and Pd), using one-pot protocols, [3 + 2] cycloaddition reactions, or sigmatropic rearrangements as Claisen’s one. 

## 2. Ring Generation via Intra-Molecular Cyclization 

### 2.1. C7a–O Bond Formation: (Route a) 

**Scheme 2:**
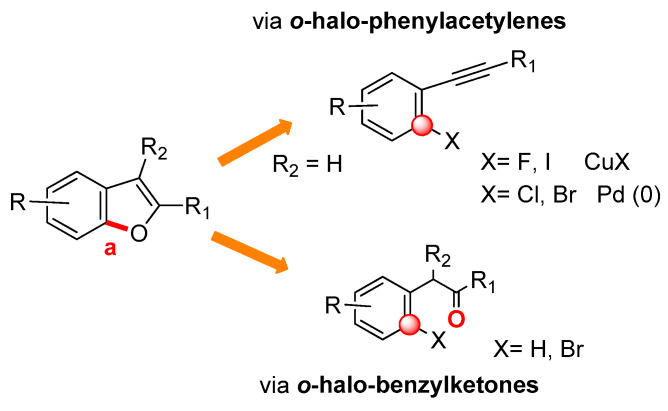
Approaches for C7a-O bond formation.

The main approaches for the formation of C7a-O bond are collected below (Scheme 2).

#### 2.1.1. From *o*-Halophenylacetylenes

An interesting alternative for in situ generating of 2-alkynylphenols is represented by the hydroxylation coupling of 2-haloarylalkynes. This strategy has been successfully employed in the domino hydroxylation–cyclization in which the conversion of 2-haloarylalkyne into a 2-phenylbenzo[*b*]furan happened in the presence of hydroxide anions.

**Scheme 3:**
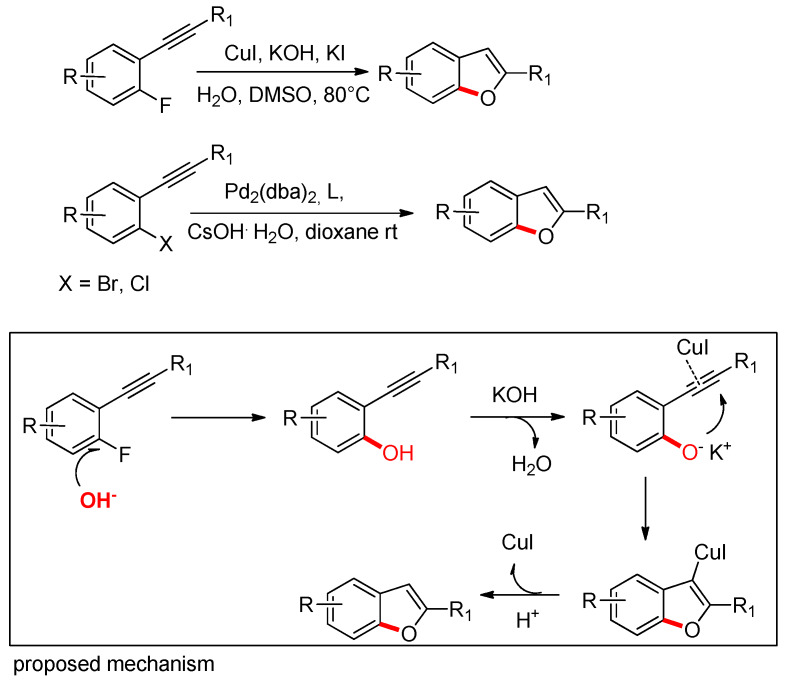
Synthesis of benzofurans via hydroxylation of *o*-halophenylacetylene.

An efficient copper-promoted hydration/annulation reaction and its application in the synthesis of benzofuran and benzothiophene derivatives has been presented, starting from readily available 2-fluorophenylacetylene derivatives (Scheme 3). This strategy involved a domino hydration of the C–F bond of 2-fluorophenylacetylene derivatives, using CuI, KOH, H_2_O, and KI in DMSO at 80 °C, followed by an intramolecular annulation to afford benzo[*b*]furan and benzo[*b*]thiophene derivatives [39]. A very similar copper-mediated hydroxylation of aryl iodide with hydroxide salts has been performed, as reported in Reference [36]. 

An extension of the hydroxylation on *o*-halide phenylacetylenes (Br and Cl) has been performed using a mixture of tris(dibenzylideneacetone)dipalladium(0) (Pd_2_dba_3_) and 5-(di-tert-butylphosphino)-1′,3′,5′-triphenyl-1′*H*-[1,4′]bipyrazole (Bippyphos), showing to be a robust and efficient catalyst system under mild conditions and with broad substrate scope (Scheme 3). Notably, a significant number of the reported reactions proceeded at room temperature, on the benchtop under air using unpurified solvents with negligible loss in reactivity vs. related transformations conducted under inert atmosphere conditions [40].

#### 2.1.2. From *o*-Halo-Benzylketones

**Scheme 4:**
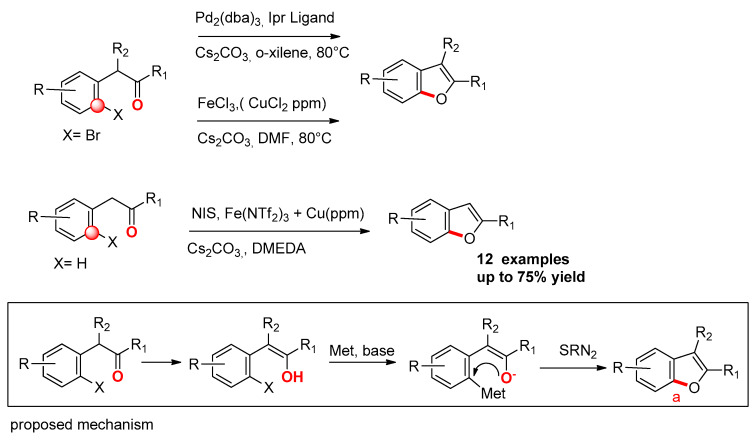
Synthesis of benzofurans from *o*-halo-benzylketones.

It was known that copper catalysts [36] were successfully applied to the synthesis of benzofurans by ring closure of aryl *o*-bromobenzyl ketones. The analogous palladium-catalyzed ring closure provided a straightforward route to 2-arylbenzofurans (Scheme 4). The best results were obtained when 1,3-bis(2,6-diisopropylphenyl) imidazolium tetrafluoroborate (IPr) was used as ligand of Pd_2_(dba)_3_, Cs_2_CO_3_ as base in *o*-xylene at 100 °C, giving nearly full conversionconversion of 2-bromobenzyl phenylketone [41]. Different aryl 2-bromobenzylketones gave rise to benzofurans if 10% mol of FeCl_3_ (of 98% or of 99.995% purity) or sub-mol % quantities of CuCl_2_ (of 99.995% purity) was used as catalyst and Cs_2_CO_3_ as base in DMF [42].

A one-pot approach for the preparation of highly substituted benzofurans was proposed, starting from simple 1-aryl- or 1-alkylketones, involving regioselective iron(III)-catalyzed halogenation, followed by metal-mediated O-arylation, as well as demonstrating the use of parts per million (ppm) copper loading to perform C−O cyclization [43]. 

A variety of benzofurans were achieved by way of a FeCl_3_-mediated intramolecular cyclization of electron-rich-aryl ketones. This method allowed the construction of benzofuran rings by linking the O-atom on the side chain to the benzene ring via direct oxidative aromatic C–O bond formation. The alkoxy substituent on the benzene ring in the substrates was essential for an efficient cyclization to occur [44].

### 2.2. O–C2 Bond Formation: (Route b)

**Scheme 5:**
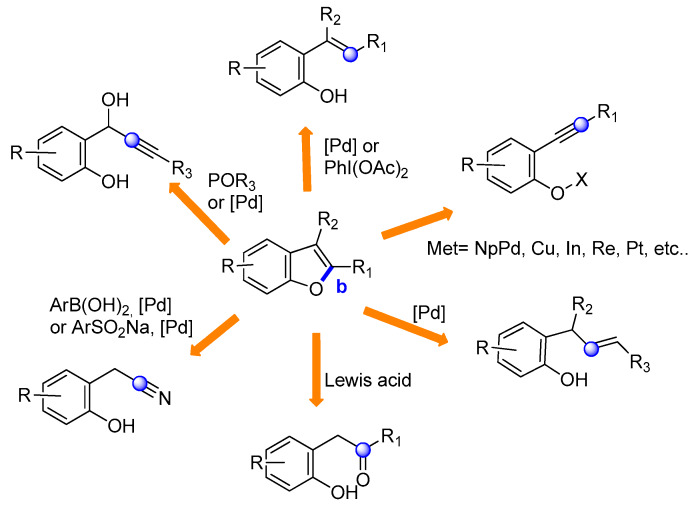
Approaches for O–C2 bond formation.

The main approaches for the formation of O-C2 bond dealt in this section are summarized below (Scheme 5).

The formation of O–C2 bond as the last bond represents the most popular between the intramolecular approaches. Several methods have been collected for this type of disconnection in the reviews of Abu-Hashem et al. [24] and Cacchi [35,36]. 

#### 2.2.1. Via C-H Activation of *o*-Alkenylphenols

**Scheme 6:**
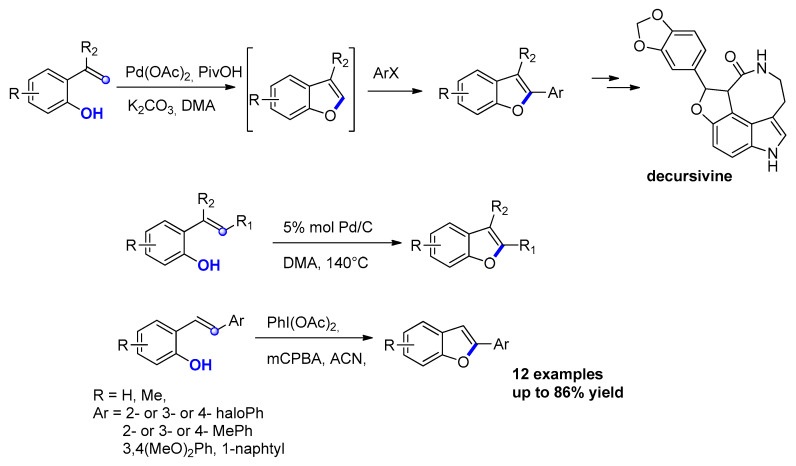
Synthesis of benzofurans via oxidative cyclization of *o*-alkenylphenols.

The C-H functionalization represents markedly a different approach from traditional ones, which exploit functional group transformations [45,46]. In 2014, a new, unprecedent palladium-catalyzed method for the synthesis of benzofurans was discovered [47] in which 2-hydroxystyrenes and iodobenzenes were involved in a C–H tandem activation/oxidation reaction. After careful analysis of the whole process, it was clear that the formation of benzofurans resulted by tandem Pd-catalyzed Heck reaction/oxidative cyclization sequence, although the detailed mechanism is still unknown. Following this method, the overall efficiency of the synthesis of decursivine and its analogues was improved (Scheme 6). 

Although significant and indicative progress has been made in the realm of oxidative C-H functionalization with stoichiometric oxidants, the C-H oxygenation involved in the one-step conversion of o-alkenylphenols to benzofurans without oxidants and sacrificing acceptors is scarcely reported. Liu and co-workers reported [48] this cyclization reaction catalyzed by palladium on carbon (Pd/C) without any oxidants and presented the perspectives of the method in the utility of ubiquitous C(sp_2_)-H bonds as latent functional groups for the construction of C(sp2)-O bonds 

An alternative route for the synthesis of 2-arylbenzofurans without the use of Pd catalyst is described by iodine(III)-catalyzed oxidative cyclization of 2-hydroxystilbenes, using 10 mol% (diacetoxyiodo)benzene [PhI(OAc)_2_] as catalyst in the presence of *m*-chloroperbenzoic acid. The 2-arylbenzofurans were isolated in good-to-excellent yields [49].

#### 2.2.2. From *o*-Alkynylphenols

Transition metal-catalyzed hydroalkoxylation of alkynes provides a reliable method for synthesizing C2-substituted benzofurans from readily available *o*-alkynylphenols [22,23,24,25,26,27,28,29,30]. Into this large sea of strategies, we report some new examples that overcome some limitations of the previous ones or represent extensions of applicability of methodologies. 

For example, a highly active heterogeneous Pd-nanoparticle catalyst for the intramolecular addition of phenols to alkynes was developed and employed in a continuous flow reaction system [50]. 

**Scheme 7:**
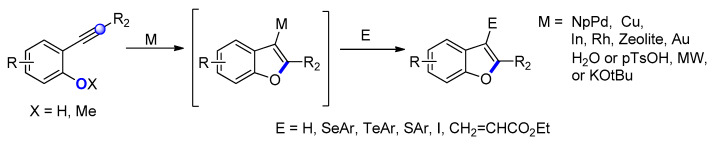
Synthesis of benzofurans via cyclization of *o*-alkynylphenols.

Indium(III) halides catalyzed the hydroalkoxylation reaction of alkynylphenols to afford benzofurans in good yields. The reaction proceeded with 5-endo-dig regioselectivity, with a variety of phenols functionalized at the arene and alkyne moieties in high yields, using InI_3_ (5 mol %) in DCE. Experimental and computational studies supported a mechanism based on the indium(III) π-Lewis acid activation of the alkyne, followed by nucleophilic addition of the phenol and final protodemetalation to afford the corresponding benzofuran [51]. Such cyclization was also reported to be efficient with Cu(I) [52], Rh(I) [53,54], Zeolite [55], Au [56], and pTsOH-MW [57], in aqueous conditions [58], or basic conditions in the last case, to afford 2-trifluoromethyl benzofurans [59] (Scheme 7). 

**Scheme 8:**
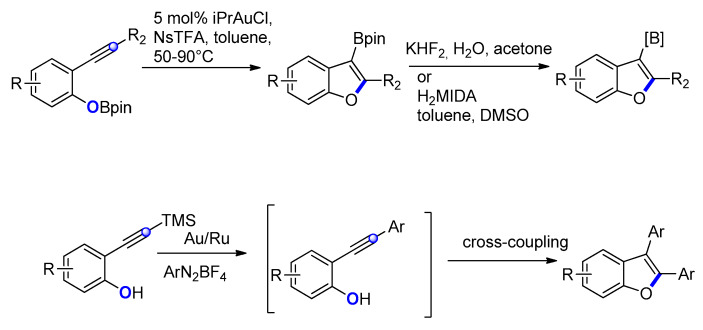
Synthesis of 2,3-disubstituted benzofurans via cyclization of *o*-alkynylphenols.

Organoboron compounds and heterocycles are powerful building blocks and precursors for organic synthesis, including for drug discovery and agrochemical and material synthesis. Blum and co-workers first studied direct oxyboration toward the formation of borylated benzofurans, where a preformed boron−oxygen σ bond was added across an alkyne activated by a carbophilic gold catalyst. Detailed mechanistic and kinetic studies of this class of reactions were reported [60,61] (Scheme 8).

A comparative study has been carried out of the catalytic activities of five-, six-, and seven membered carbene complexes [(NHC)AuX], [(Ph_3_P)AuX], and [(Me_2_S)AuX], and inorganic compounds of gold in model reactions of indole and benzofuran synthesis. A selective and convenient synthesis of 2,3-diarylbenzofurans has been developed with the aid of light and taking advantage of a cooperative gold/photoredox-catalyzed two-fold arylation reaction of TMS-terminated alkynols. The photoredox sequence involving 2-[(trimethylsilyl)ethynyl]phenol exclusively afforded 2,3-diarylbenzofurans. The reaction of terminal alkynes proceeded in poor yields, while the use of bulkier silyl groups, such as TIPS, resulted unproductive. Apparently, the C(sp) arylation reaction was the first event on the domino *bis*-arylative sequence. These results could be explained through the intermediation of arylgold(III) species and several single electron transfer processes [62] (Scheme 8).

**Scheme 9:**
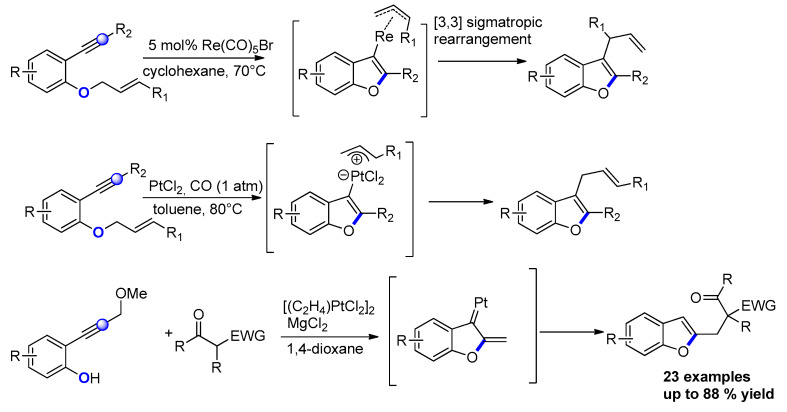
Synthesis of benzofurans via cyclization of *o*-alkynylphenyl allyl ethers.

A rhenium-catalyzed carboalkoxylation and carboamination of alkyne was reported by Zi and co-workers, providing an efficient route to synthesize de novo C3-substituted benzofurans and indoles under mild conditions in moderate-to-good yields [63]. Mechanistic studies revealed that the rhenium played the role of a π acid catalyst to activate the alkynes, followed by a charge-accelerated [3,3]-sigmatropic rearrangement. An analogue activation of the alkyne was proved by the electrophilic Pt-species, which enables nucleophilic attack by the heteroelement, resulting in *trans* alkoxyplatination [64]. This process formally generates an allyl cation that reacts to the most nucleophilic position of the ring to give the product and regenerate the catalyst. This notion suggests that other entities R_1_, which are able to stabilize positive charge, might transfer in a similar fashion. Moreover, Ferreira’s group investigated on the use of platinum catalysis to generate α,β-unsaturated carbene intermediates via an intramolecular nucleophilic addition into alkynes bearing propargylic ethers. These carbenes have been demonstrated to undergo cycloadditions, hydrogen migrations, and vinylogous nucleophilic additions [65], where β-diketones, ketoesters, and ketoamides all successfully added onto the platinum carbene intermediate (Scheme 9).

#### 2.2.3. From *o*-Gem-Dibromoalkenyl Phenols

**Scheme 10:**
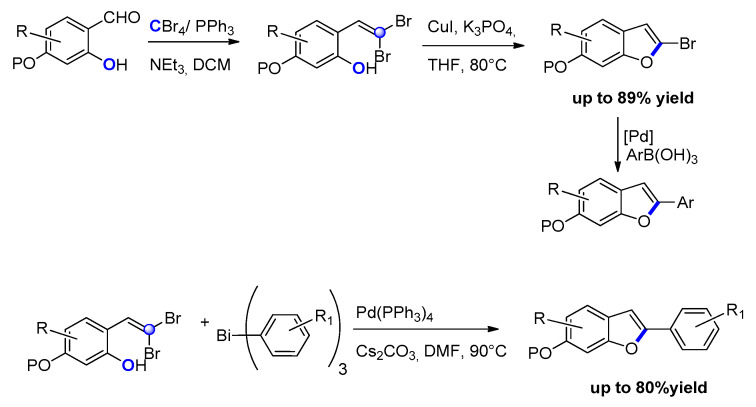
Synthesis of benzofurans via o-gem-dibromoalkenyl phenols.

The popular strategy involving a Sonogashira coupling/cyclization protocol, using ethoxycarbonylethenyl-derived 2-halophenols and alkynes, worked well for certain compounds but failed, for example, for egonol and other related compounds. This failure was attributed to the electronic nature of both the 2-halophenols and the alkynes, and the related complications when applied in a multistep synthesis. Thus, this protocol did not facilitate a rapid and concise synthesis of benzofuran natural products. A seminal strategy was reported by Lautens et al., in 2009, in which 2-bromobenzofurans were generated by a Cu-catalyzed cyclization of 2-(2,2-dibromovinyl)-phenols [66]. The same approach was used by Kim’s group, to synthesize different natural products [67,68], while recently, Rao et al. explored the application of a Pd-catalysed domino cyclization/coupling protocol in a pooled approach for the synthesis of benzofuran natural products [69,70] (Scheme 10).

#### 2.2.4. From *o*-Allylphenols

**Scheme 11:**
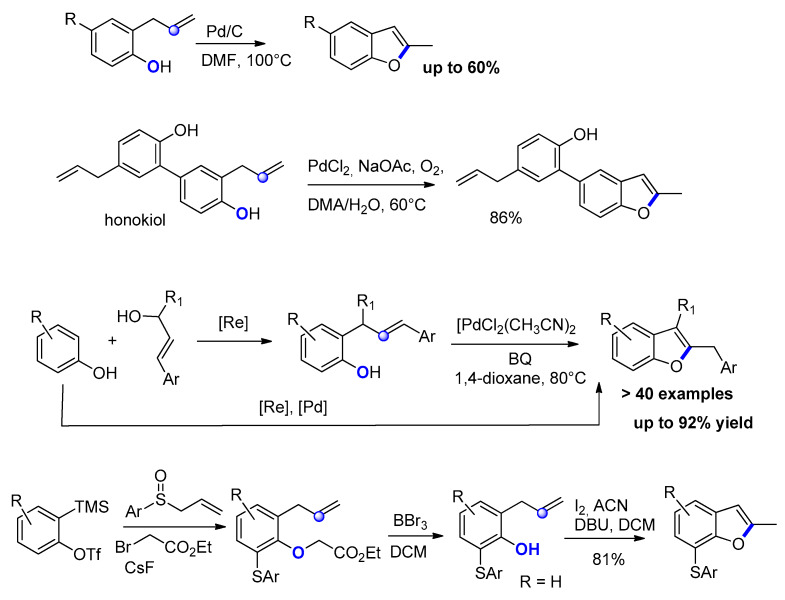
Synthesis of bezofurans via cyclization of *o*-allyl phenols.

A special position in palladium catalysis constitutes palladium on carbon (Pd/C). Palladium on carbon (Pd/C) has some unique advantages, like stability in air, easy removal by simple filtration, sustainability, and commercial availability at a relatively low cost [71]. Kokotos and co-workers described a cheap and easy-to-execute strategy for the synthesis of benzofurans, employing Pd/C as the promoter. A variety of substituted allyl-phenols were converted into the desired products in good to excellent yields. Recycling of Pd/C was possible up to five cycles, keeping similar levels of reactivity [72] (Scheme 11). From the natural product honokiol, which contains a *o*-allylphenol fragment, a benzofuran scaffold was produced through a Wacker-type intramolecular cyclization, using PdCl_2_, NaOAc, and O_2_, in DMA/H_2_O [73]. Different substituted *o*-allylphenol derivatives, prepared via a Friedel−Crafts alkylation of cinnamyl alcohol with phenols, using Re_2_O_7_ catalyst in acetonitrile as solvent, underwent oxidative cyclization, using PdCl_2_(C_2_H_4_)_2_ as catalyst and BQ as oxidant [74]. A simple sequential reaction protocol has been developed for the synthesis of functionalized 2-benzyl benzofurans via Friedel−Crafts alkylation of phenols with cinnamyl alcohols in the presence of Re_2_O_7_ catalyst, followed by Pd(II)-catalyzed oxidative annulation of in situ generated *o*-cinnamyl phenols. Synthesis of 2-benzyl benzofurans was achieved in good yields (disconnection **b**+**d**). In the work of Li’s group, the *o*-allylphenol derivatives were generated from aryne, using Kobayashi benzyne precursor, and aryl allyl sulfoxide [75] (Scheme 11).

#### 2.2.5. From *o*-Hydroxybenzyl Ketones

**Scheme 12:**
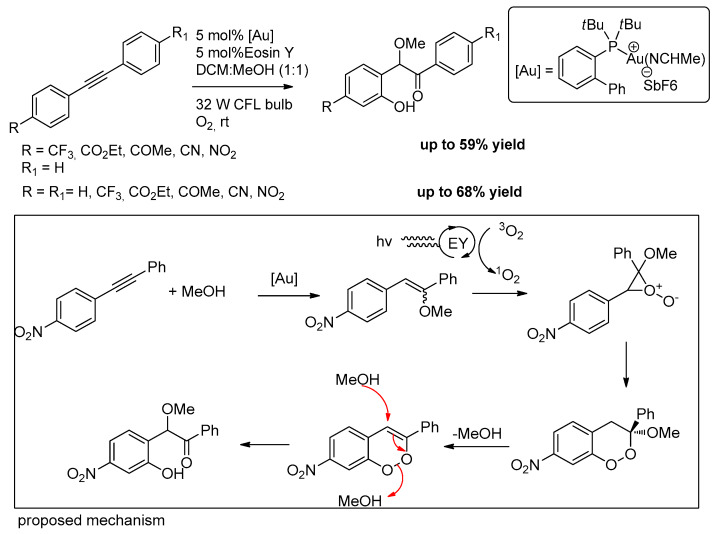
Synthesis of benzofurans via *o*-hydroxybenzyl ketones.

A new method to generate *o*-hydroxybenzyl ketones was proposed recently by Patil and co-workers, using an *o*-oxygenative 1,2-difunctionalization of diarylalkynes. This procedure allowed under merged gold/organophotoredox catalysis to access highly functionalized 2-(2-hydroxyaryl)-2-alkoxy-1-arylethan-1-ones [76]. Detailed mechanistic studies suggested a relay process, initiating with gold-catalyzed hydroalkoxylation of alkynes, to generate enol-ether followed by a key formal [4 + 2]-cycloaddition reaction. This type of oxidation of alkynes depended strongly on the nature of substituents on the aryl. Symmetrical diarylalkynes with electron withdrawing groups gave rise to the corresponding α-methoxyketones with yield up to 68%, while with asymmetrical alkyne lower yields were obtained. Not all the functionalities were well tolerated. The successful application of the present methodology was also shown for the synthesis of benzofurans (Scheme 12).

**Scheme 13:**
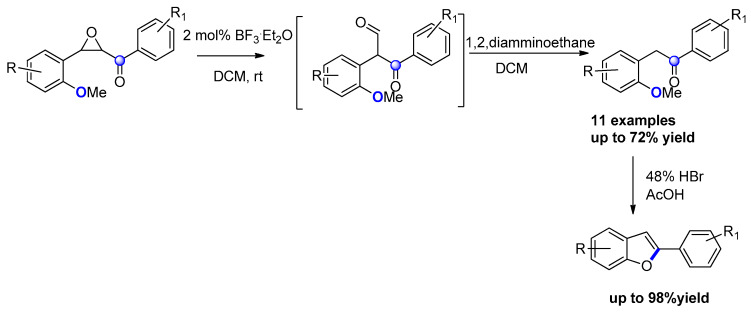
Synthesis of benzofurans via *o*-methoxychalcone epoxides.

An efficient and practical method for construction of 2-arylbenzofurans from 2-methoxychalcone epoxides has been reported. Catalyzed by 2 mol % of BF_3_·Et_2_O, 2-methoxychalcone epoxides went through the Meerwein rearrangement, followed by deformylation in one-pot to successfully afforded 2-methoxydeoxybenzoins. Afterward, 2-arylbenzofurans were obtained in high yields (87–100%) via inter-molecular cyclodehydration of 2-methoxydeoxybenzoins with 48% HBr [77] (Scheme 13).

#### 2.2.6. From *o*-(Cyanomethyl) Phenols

**Scheme 14:**
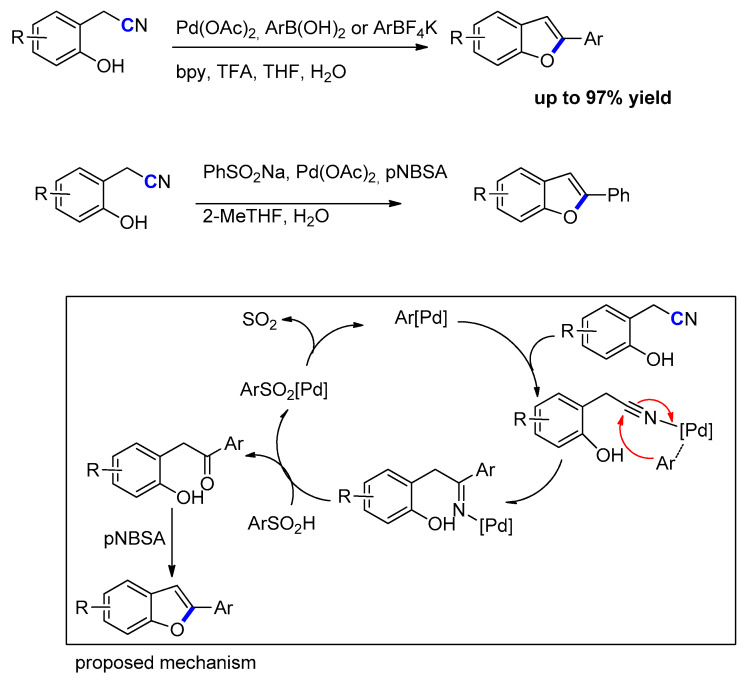
Synthesis of benzofurans via *o*-(cyanomethyl) phenols.

A seminal study for exploring the potential of palladium-catalyzed addition of potassium organotrifluoroborates to a nitrile group, which is usually more challenging than an aldehyde or acyl chloride group, has been performed by Whu’s group [78,79]. This work has provided a new method for the synthesis of alkyl aryl ketones (or dicarbonyl compounds) by Pd-catalyzed addition of arylboronic acid or potassium aryltrifluoroborates to aliphatic nitriles (or dinitriles) and the one-step synthesis of 2-arylbenzofuran derivatives (Scheme 14).

The first example of the palladium-catalyzed one-pot synthesis of 2-arylbenzofurans in moderate to excellent yields via a tandem reaction of 2-hydroxyarylacetonitriles with sodium sulfinates was reported in 2014. A plausible mechanism for the formation of 2-arylbenzofurans involving desulfinative addition and intramolecular annulation reactions is proposed. Moreover, the present synthetic route to benzofurans could be readily scaled up to the gram quantity, without any difficulty. Thus, the method represents a convenient and practical strategy for synthesis of benzofuran derivatives [80,81].

#### 2.2.7. From 1-(2-hydroxyphenyl) Propargyl Alcohol Derivatives 

**Scheme 15:**
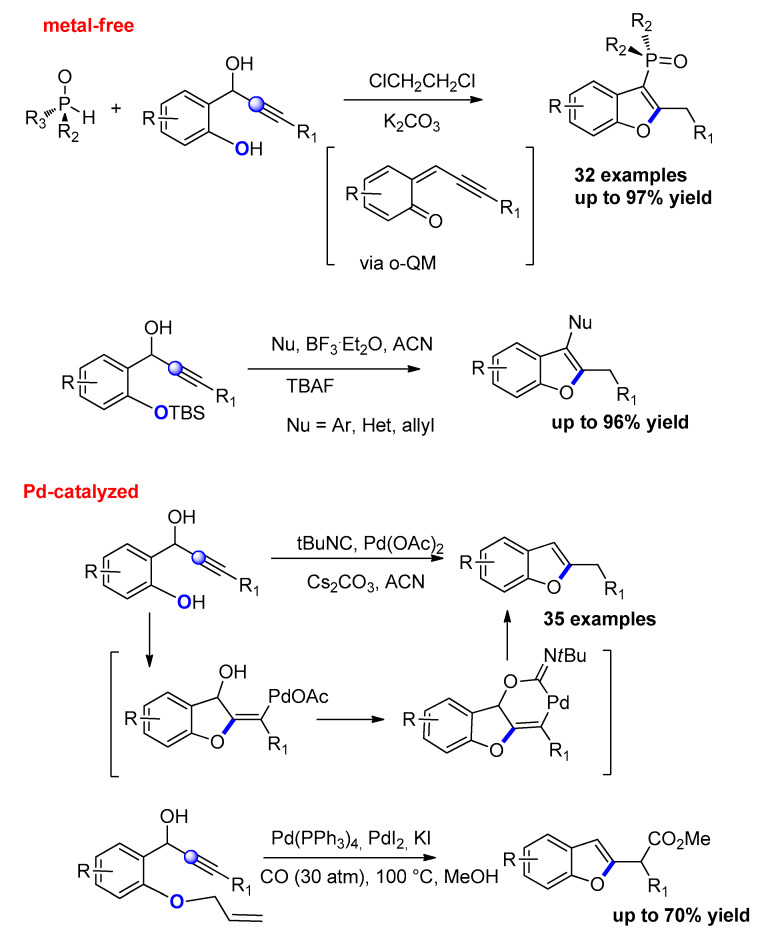
Synthesis of benzofurans via 1-(2-hydroxyphenyl)propargyl alcohol derivatives.

A novel metal-free one-pot protocol for the effective and efficient synthesis of 3-phosphinoylbenzofurans via a phospha-Michael addition/cyclization of H-phosphine oxides and in situ generated *o*-quinone methides was described. Based on the expeditious construction of C(sp2)−P bonds, asymmetric synthesis of optically pure 3-phosphinoylbenzofurans containing chiral P-stereogenic center has also been probed by using chiral RP-(−)-menthyl phenylphosphine oxide [82]. A metal-free procedure used BF_3_-Et_2_O to catalyze the cyclization and the substitution with a nucleophle [83]. Moreover, Pd-catalyzed cyclization was reported on these substrates [84,85] (Scheme 15).

### 2.3. C2–C3 Bond Formation: (Route c)

**Scheme 16:**
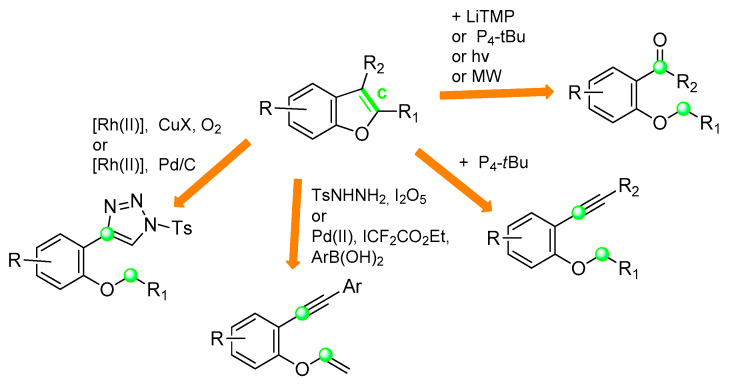
Approaches for C2–C3 bond formation.

The main approaches for the formation of C3-C2 bond dealt in this section are summarized below (Scheme 16).

#### 2.3.1. From *o*-(Alkoxy)Phenyl Arylketones

**Scheme 17:**
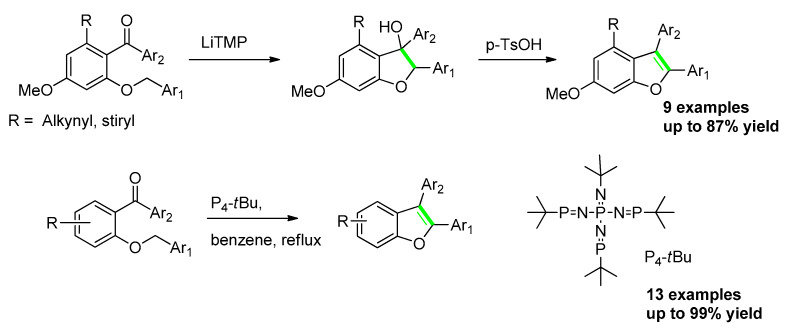
Synthesis of benzofurans via *o*-benzyloxyphenyl arylketones.

Dehydrative decarboxylation of *o*-acylphenoxyacetic acids or esters on treatment with a base represents an old methodology to prepare benzofurans. A more recent approach used benzylic deprotonation, using LiTMP, followed by an intramolecular cyclization between the carbanion and carbonyl group of the corresponding arylketone and subsequent dehydration acid catalyzed (*p*-TsOH·H_2_O), to deliver benzofuran, the core structure for the synthesis of the natural and biologically relevant products, Malibatol A and Shoreaphenol (Scheme 17) [86].

Moreover, 2,3-Diarylbenzofurans were also efficiently generated by the cyclization of *o*-benzyloxybenzophenones, using the hindered phosphazene base P_4_-*t*-Bu [87].

**Scheme 18:**
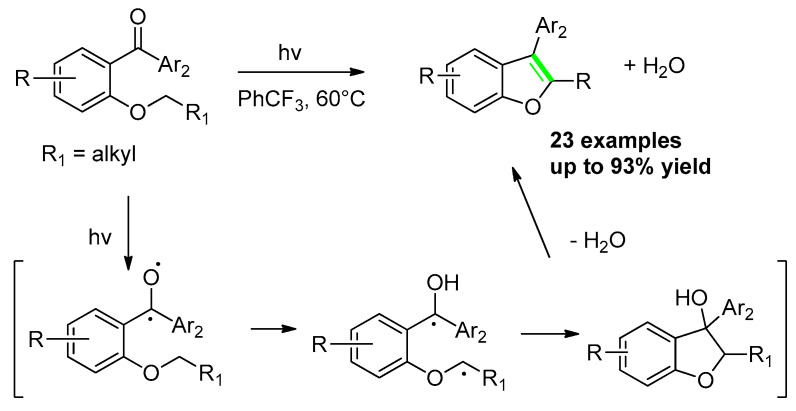
Synthesis of benzofurans via *o*-alkoxyphenyl arylketones.

Condensation of carbonyls with non-acidic methylenes such as those adjacent to heteroatoms and allylic types to generate C=C bonds is challenging but highly desirable. An advanced method overcame this limitation. Li and co-workers reported a simple, clean, and high-yielding protocol promoted by UV-light, to achieve condensation of non-acidic methylenes with carbonyls. As examples to demonstrate the power of this methodology, benzofurans were synthesized with broad functional group compatibility [88] (Scheme 18). 

#### 2.3.2. From *o*-Alkynylphenyl benzyl (or Allyl)Ethers

**Scheme 19:**
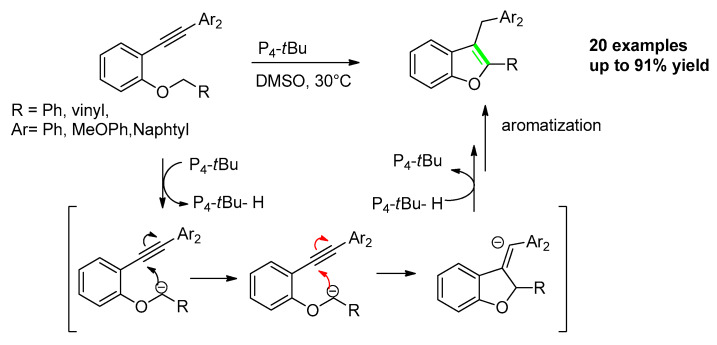
Synthesis of benzofurans via *o*-alkynylphenyl ethers.

Terada group demonstrated that the organic superbase phosphazene P_4_-*t*Bu worked as an active catalyst for intramolecular cyclization of *o*-alkynylphenylethers, reporting the carbon–carbon bond formation under mild reaction conditions without the need for a metal catalyst, providing an efficient synthetic method for 2,3-disubstituted benzofurans derivatives [89] (Scheme 19).

#### 2.3.3. From *o*-Alkynylphenyl Vinylethers

**Scheme 20:**
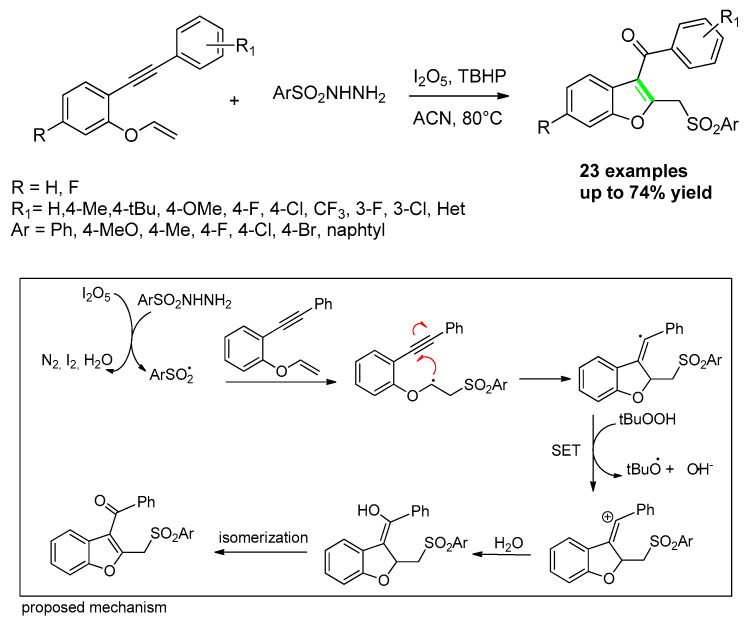
Synthesis of benzofurans via *o*-alkynylphenyl vinylethers.

A simple I_2_O_5_-mediated method has been developed under metal-free conditions for the construction of sulfonylated benzofurans. The present reaction was efficiently achieved through the oxidative cyclization of 1,6-enynes and arylsulfonylhydrazides, which provided an attractive approach to a series of sulfonylated benzofurans in moderate to good yields [90] (Scheme 20).

**Scheme 21:**
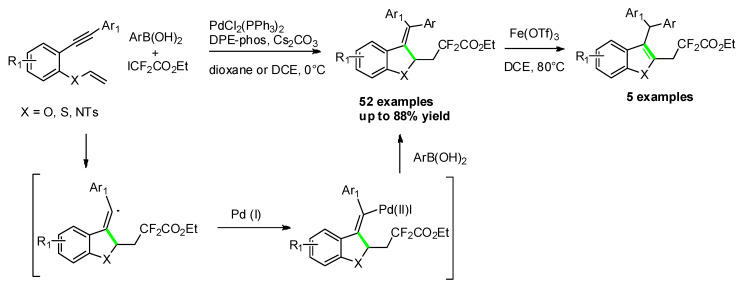
Synthesis of benzofurans via *o*-alkynylphenyl vinylethers.

Very recently, the same substrates were used by Zhang’s group to introduce difluoroalkylated substituent on benzofurans, according to their expertise in the transition-metal catalyzed cross-coupling of difluoroalkylhalide and boronic acids. Difluoroalkylated benzofuran derivatives were constructed via palladium-catalyzed cascade difluoroalkylation-arylation of 1,6-enyne. Moreover, final difluoroalkylated benzofurans were obtained through an isomerization process catalyzed by Fe(OTf)_3_ [91] (Scheme 21).

**Scheme 22:**
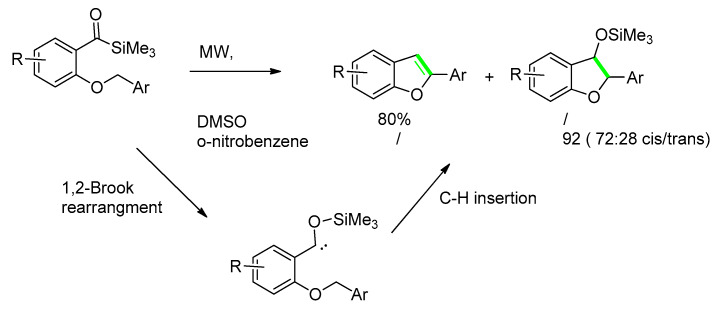
Synthesis of benzofurans via *o*-acylsylanesphenyl benzyl ethers.

Within the broad field of C-H bond functionalization, the insertion of carbenes into C-H bonds is arguably the best approach for directly transforming a C-H bond directly into a C-C bond. Dong strategy [92] for C-H bond functionalization was inspired by Adrian Brook’s discovery of the unique ability of acylsilanes to undergo thermal and photochemically induced 1,2 silicon-to-oxygen migration [93]. This Brook rearrangement of acylsilanes could be considered an umpolung process, where the acylsilanes acted as a carbonyl anion equivalents. Thus, a thermally induced Brook rearrangement generated a transient siloxycarbene that underwent to a rapid insertion into a neighboring C-H bond (Scheme 22). Thus, this new approach furnished 2,3-dihydrobenzofuran and benzofuran derivatives under microwave irradiation, in which the solvent played an important role to determine the generated species. 

#### 2.3.4. From *o*-Triazole-Phenyl Benzylethers

**Scheme 23:**
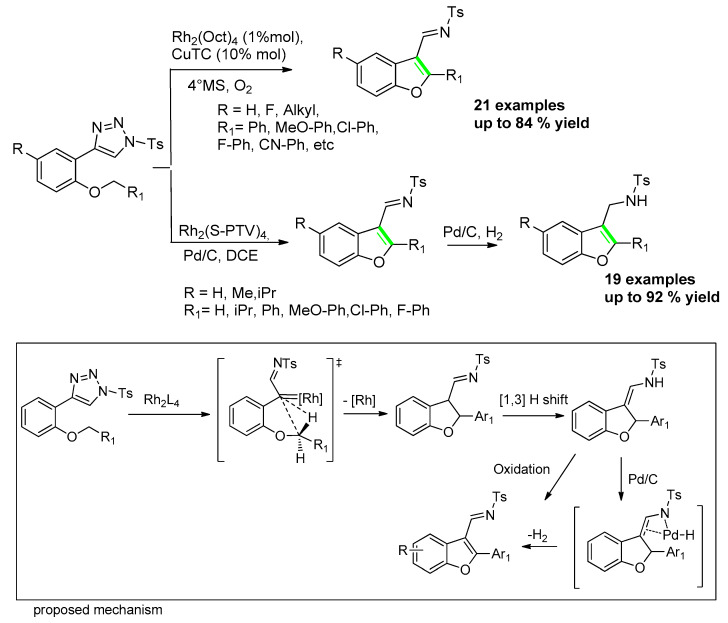
Synthesis of benzofurans via *o*-*N*-sulfonyl1,2,3-triazolphenyl bezylethers.

Recently, *N*-sulfonyl-1,2,3-triazoles have emerged as alternative precursors for the formation of metallocarbenes [94]. Independent studies from Kang [95] and Chen [96] reported intramolecular sp_3_ C–H insertion reaction of α-imino rhodium carbene generated from *N*-sulfonyl-1,2,3-triazoles. The first one reported the use of O_2_ as the oxidant to obtain benzofurans, whereas the second one used Pd/C, to allow the isomerization of the allylic portion of enamine, which, in presence of H_2,_ afforded to amine derivatives. Both methodologies furnished a number of benzofuran derivatives in good to excellent yields (Scheme 23).

### 2.4. C3–C3a Bond Formation: (Route d)

**Scheme 24:**
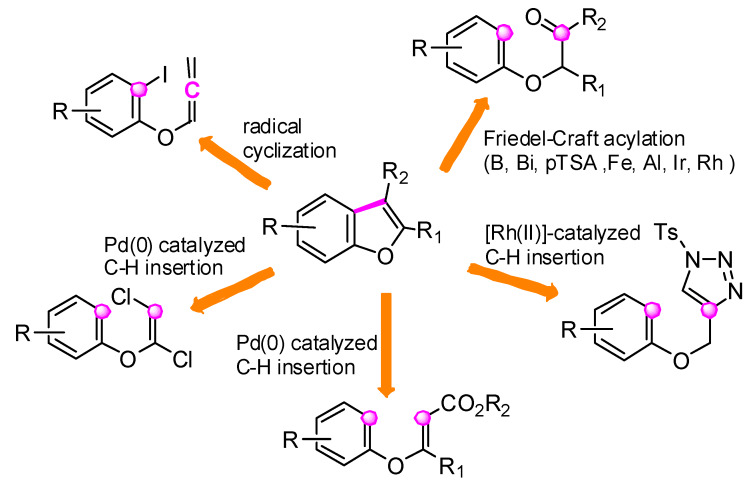
Approaches for the C3–C3a bond formation.

The main approaches for the formation of C3-C3a bond dealt in this section are summarized below (Scheme 24).

#### 2.4.1. Via Friedel–Crafts Acylation 

**Scheme 25:**
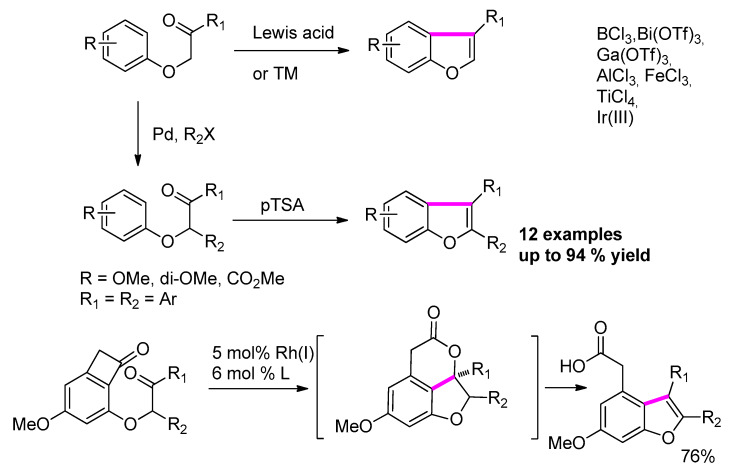
Synthesis of benzofurans via Friedel–Crafts acylation.

Outstanding total synthesis of multisubstituted benzofurans were achieved by intra-molecular Friedel–Crafts acylation of α-aryloxyaryl ketones, which were prepared from an inter-molecular *O*-alkylation of α-haloarylketones with phenoxide. Many examples of Lewis acid and transition-metal-catalyzed direct intramolecular cyclodehydration of the resulting α-aryloxyaryl ketones have been developed by several research groups. Kim’s group used BBl_3_ [97], pTSA [98], or Bi(OTf)_3_ [99] in the total synthesis of natural stilbenoids, while Chang’s group used Ga(OTf)_3_ [100] for the first time in such cyclization. Arava’s group was concerned with AlCl_3_ or FeCl_3_ [101], while Tang investigated on TiCl_4_ [102]. Shibata and co-workers used In(III) species generated from [CpIrCl_2_]_2_ and AgSbF_6_, in which the presence of the acetyl group on the aryl group allowed the Ir insertion in C-H bond [103]. Recently, Xu, employing 10 mol% [Rh(cod)(MeCN)_2_]BF_4_ and 12 mol% DPPF in THF, developed a cascade transformation initiated by regioselective activation of benzocyclobutenone, followed by insertion into C=O and spontaneous aromatization, which generated 2,3-disubstituted benzofurans [104] (Scheme 25). 

#### 2.4.2. Via [Ru(II)] C-H Insertion of *N*-Sulfonyl-1,2,3-Triazole Derivatives 

**Scheme 26:**
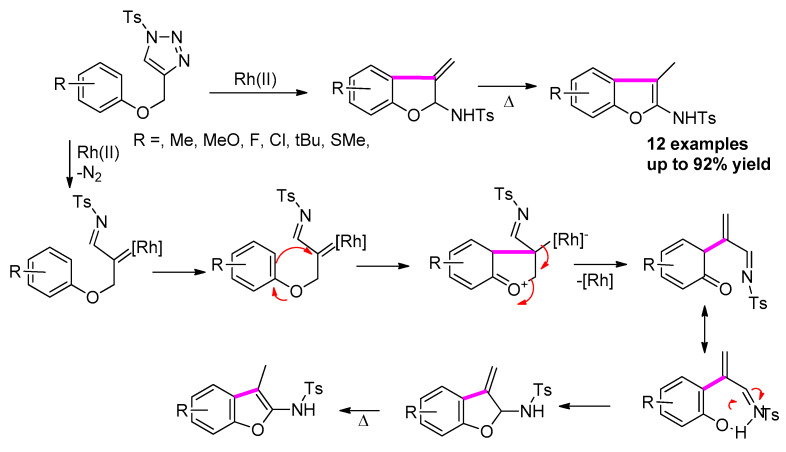
Synthesis of benzofurans via [Rh(II)] C–H insertion of *N*-sulfonyl-1,2,3-triazol derivatives.

The selective synthesis of substituted 3-methylene-2,3-dihydrobenzofurans and 3-methylbenzofurans was developed in the seminal work of Shi through Rh(II)-catalyzed denitrogenative annulation of *N*-sulfonyl-1,2,3-triazole at ambient to mild heating condition, respectively [105] (Scheme 26). Further, a one-pot strategy was also developed by using substituted *O*-propargylphenols through Cu/Rh-catalyzed method, to rapidly construct 3-methylene-2,3-dihydrobenzofuran derivatives [106].

#### 2.4.3. Via [Pd(0)] C–H Insertion

In 2008, Hultin’s group reported the synthesis of 2-substituted benzofurans from simple phenols, boronic acids or other organoboron reagents, and trichloroethylene. The overall process required only two synthetic steps, with the key step being a one-pot sequential Pd-catalyzed Suzuki cross-coupling/direct arylation reaction. The method tolerated many useful functional groups and did not require the installation of any other activating functionality [107,108]. 

**Scheme 27:**
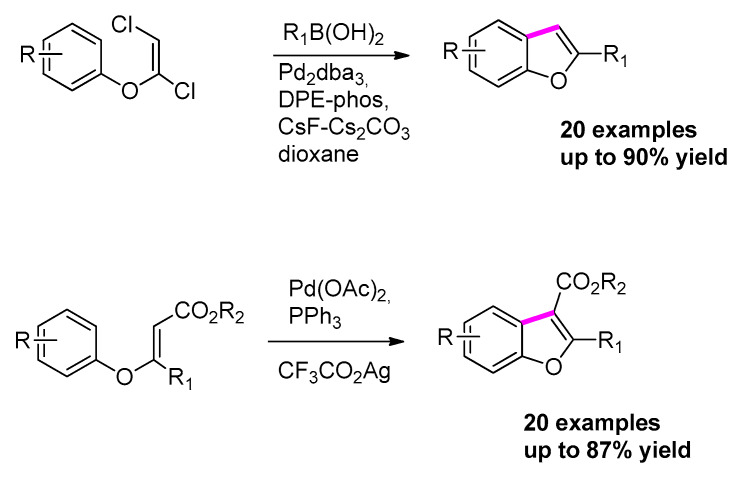
Synthesis of benzofurans via [Pd (0)] C–H insertion of vinyl derivatives.

In 2011, Wang and co-workers developed a synthesis of benzofurans from commercially available phenols and propiolate through the direct oxidative cyclization. In the presence of Pd(OAc)_2/_PPh_3_ and CF_3_CO_2_Ag, (*E*)-type 3-phenoxyacrylates underwent reaction smoothly to generate the corresponding benzofurans in good yields in benzene at 110 °C, with no need for an inert atmosphere. This transformation of phenols into benzofurans was also carried out in one-pot, in a simple and efficient way [109] (Scheme 27).

#### 2.4.4. Via Radical Cyclization of *o*-Iodophenyl Allenyl Ethers

**Scheme 28:**
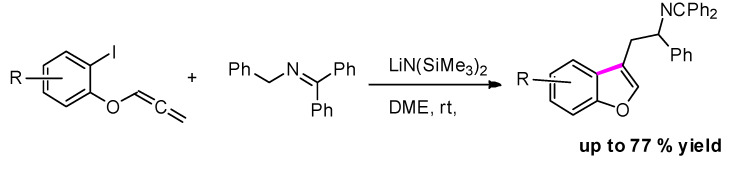
Synthesis of benzofurans via *o*-iodophenyl allenylethers.

Recently, a mild and broadly applicable methodology to prepare complex benzofurylethylamine derivatives through a unique radical cyclization cascade mechanism was reported [110]. Single-electron transfer (SET) from 2-azaallyl anions to 2-iodo aryl allenyl ethers initiated a radical cyclization that was followed by intermolecular radical–radical coupling. A diverse series of benzofurylethylamine derivatives was prepared in good-to-excellent yields, in three steps, from 2-iodophenols (Scheme 28). This methodology could also be extended to build larger heterocycles.

## 3. Ring Generation via Intermolecular Cyclization

### 3.1. C7a–O and C3–C3a Bond Formation: (Route a + d) 

**Scheme 29:**
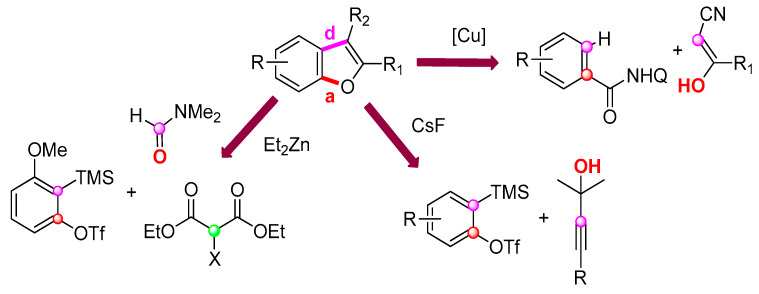
Approaches for C7a-O and C3–C3a bond formation.

The main approaches for the formation of C7a-O and C3-C3a bond dealt in this section are summarized below (Scheme 29).

#### 3.1.1. Via *o*-C-H alkylation/Decarboxylation

**Scheme 30:**
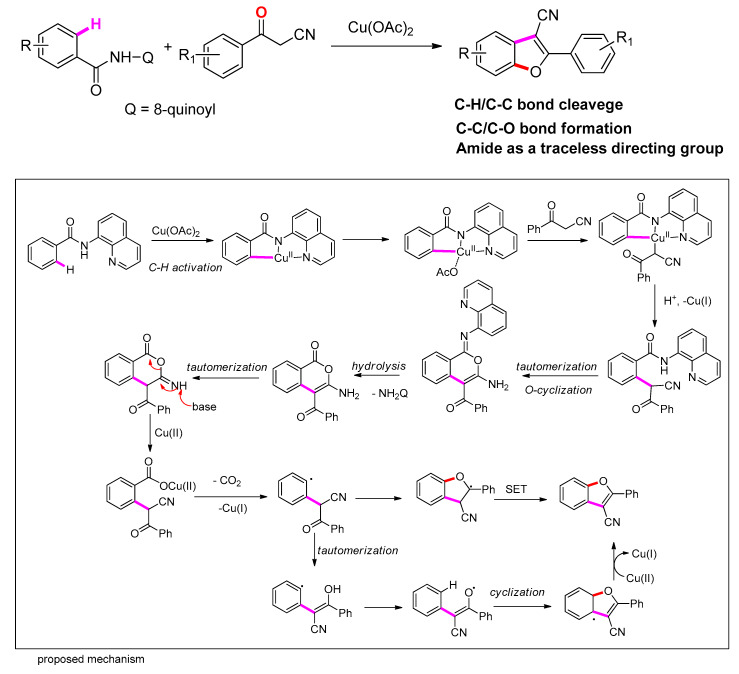
Synthesis of benzofurans via C-H alkylation/decarboxylation.

Transition-metal-catalyzed directing-group-assisted C-H bond functionalization has proven to be a powerful strategy for the construction of carbon–carbon and carbon–heteroatom bonds because of its great potential for step-economy and environmental sustainability. A range of carboxyl directed *o*-C-H alkylation/decarboxylation reactions [111,112,113,114,115] has been exploited. In all transformations, the directing group plays the key role for reaction efficiency and regioselectivity. However, the installation and disconnection of a directing group require extra and tricky steps, which determine severe limitations on application. A very recent copper-mediated synthesis of 2,3-disubstituted benzofurans from readly available benzamides and benzoylacetonitriles was described, in which the assistance of an 8-aminoquinolyl auxiliary was shown [116]. To overcome the above shortcomings, carboxyl group was successfully used as a traceless directing group, which could introduce a target functional group into a specific position of the substrate and then be completely removed by decarboxylation. In this strategy, the C3–C3a bond was successfully constructed via C–H activation, and C7a–O bond was subsequently formed at the original position of the amide group in a one-pot manner. The amide directing group was removed simultaneously under the reaction conditions through C–C bond cleavage (Scheme 30). 

#### 3.1.2. Via Propargyl Claisen Rearrangement/Cycloaddition

**Scheme 31:**
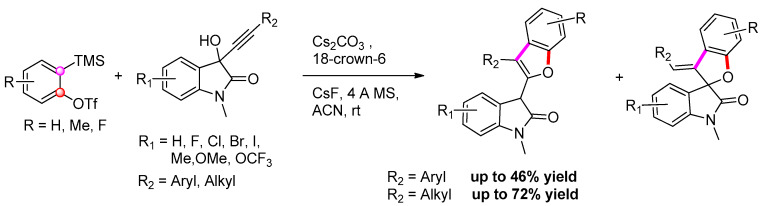
Synthesis of benzofurans via propargyl Claisen rearrangement/cycladdition.

Synthetic methodologies for the preparation of benzofuran derivatives through transition-metal/noble-metal catalysts, Lewis/Brønsted acids, and base-promoted cyclizations reported propargylic alcohols or their derivatives as starting materials. However, accessing such preferred scaffolds using aryne chemistry is less known. Palakodety and co-workers [117] reported an unprecedented base-mediated cyclization of propargylic alcohols with aryne, providing a novel method for the synthesis of 3-benzofuryl-2-oxindole and 3-spirooxindole benzofuran scaffolds via a propargyl Claisen rearrangement/cycloaddition pathway (Scheme 31). The nature of the substituent on acetylene group of propargylic alcohol influenced the outcome of the reaction. The protocol offered a transition-metal-free and operationally simple methodology with broad substrate scope as a ready access to complex oxindole-linked heterocyclic compounds. 

#### 3.1.3. Via Addition of Zinc-Enolate to Methines

**Scheme 32:**
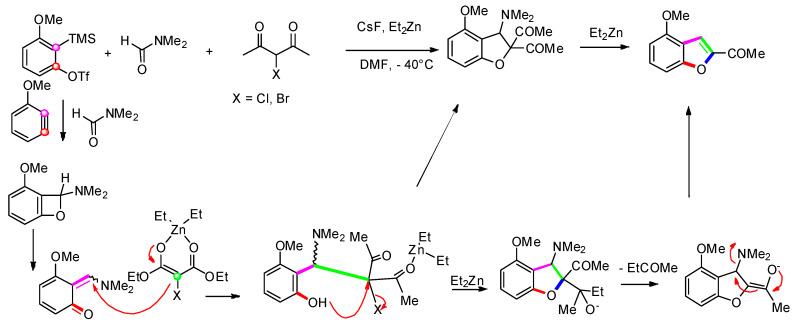
Synthesis of benzofurans via addition of zinc-enolate to methynes.

Miyabe’s group developed an efficient insertion of arynes, which were generated in situ from *o*-(trimethylsilyl)aryl triflates and the fluoride ion, into the C=O π-bond of formamides. The subsequent addition of zinc enolates of α-chlorinated methines gave rise to benzofurans, via the addition of an ethyl anion to the dihydrobenzofurans bearing a ketone group and the retro-aldolic process inducted by Et_2_Zn [118] (Scheme 32). 

### 3.2. O–C2 and C2–C3 Bond Formation: (Route b + c)

**Scheme 33:**
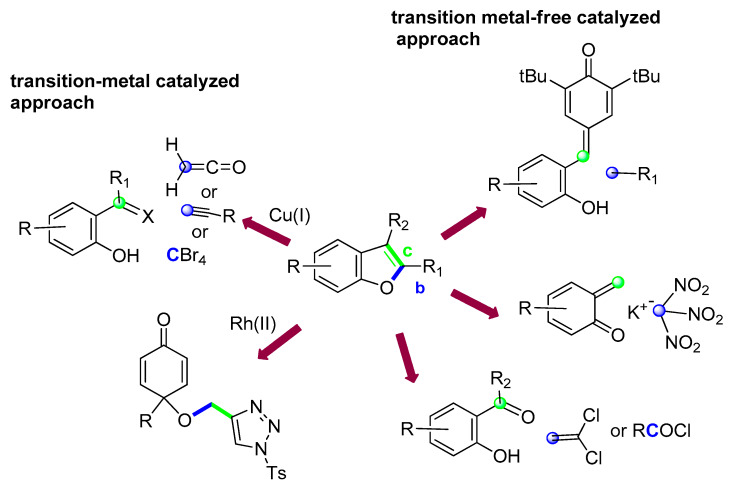
Approaches for the O–C2 and C2–C3 bond formation.

The main approaches for the formation of O–C2 and C2–C3 bond dealt in this section are summarized below (Scheme 33).

#### 3.2.1. Via Transition-Metal-Free Catalyzed Approaches: *p*-Quinone Methides

Recently, *p*-quinone methides (*p*-QMs) were subjected to extensive investigation for their interesting chemical properties. A typical reaction of *p*-QMs involves rearomatization via nucleophilic addition by a variety of carbon nucleophiles.

**Scheme 34:**
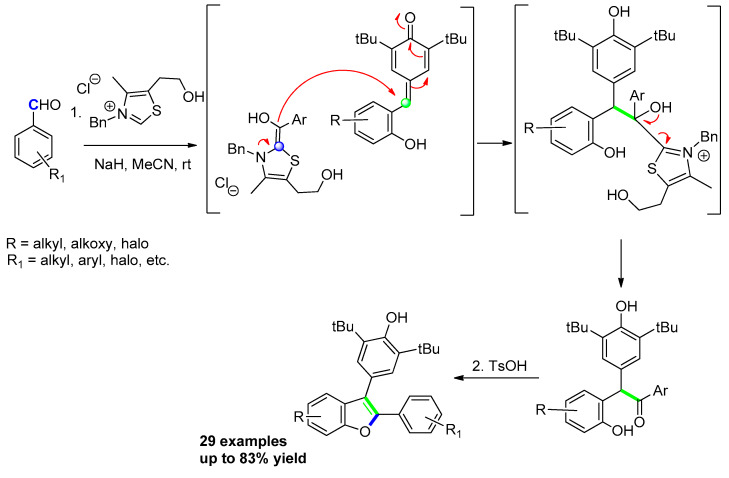
Synthesis of benzofurans via addition of aromatic aldehyde to *p*-quinone methide.

A one-pot protocol for the synthesis of 2,3-diarylbenzo[b]furan derivatives through an *N*-heterocyclic carbine catalyzed 1,6-conjugate addition of aromatic aldehydes to 2-hydroxyphenyl-substituted *p*-quinone methides followed by acid-mediated dehydrative annulation has been developed. This protocol allows access to a wide range of 2,3-diarylbenzofuran derivatives in moderate-to-good yields [119] (Scheme 34).

**Scheme 35:**
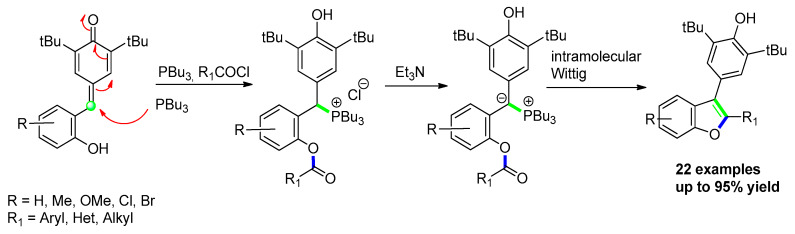
Synthesis of benzofurans via addition of phosphines to *p*-quinone methides.

In this context, an efficient synthesis of functionalized benzofurans was obtained under mild and metal-free conditions from the *p*-QMs bearing an *o*-hydroxy group, treated with phosphine, acyl chloride, and base. Through a 1,6-phospha-Michael addition, O-acylation, and subsequent Wittig pathway, this protocol was demonstrated to be useful for the synthesis of benzofurans [120] (Scheme 35).

#### 3.2.2. Via transition-Metal-Free Catalyzed Approaches: *o*-Quinone Methides

**Scheme 36:**
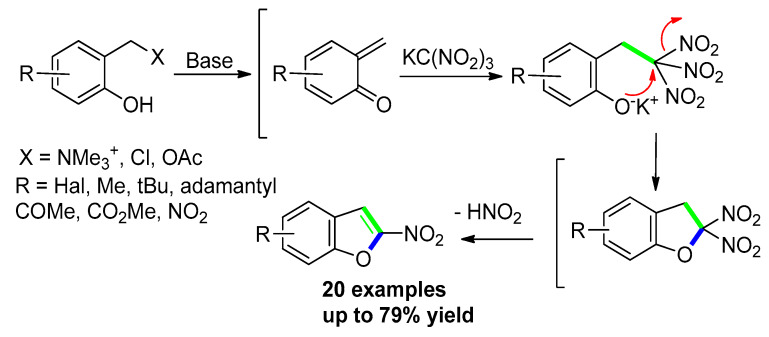
Synthesis of benzofurans via addition of trinitromethanide to *o*-quinone methides.

*o*-Quinone methides (*o*-QMs) are highly reactive and useful species that have been implicated in the reaction with nucleophiles as 1,4-Michael acceptors [121,122]. Moreover, ambiphilic synthons, which contain both electrophilic and nucleophilic centers in the same molecule, are widely used in organic synthesis as useful building blocks. The development of new synthetic methods using ambiphiles has great potential in the elaboration of new high-step-economy reactions. The first example of the use of potassium trinitromethanide as a 1,1-ambiphilic synthon equivalent for the construction of a benzofuran moiety, mediated by triethylamine, has been developed. The method tolerates a variety of functional groups on the starting quaternary ammonium salt and has been successfully extended to polysubstituted benzofurans. The formation of an *o*-quinone methide intermediate is postulated as a key step in this cascade process [123] (Scheme 36).

#### 3.2.3. Via Transition-Metal-Free Catalyzed Approaches: *o*-Hydroxyphenone or Salicylaldehydes

**Scheme 37:**
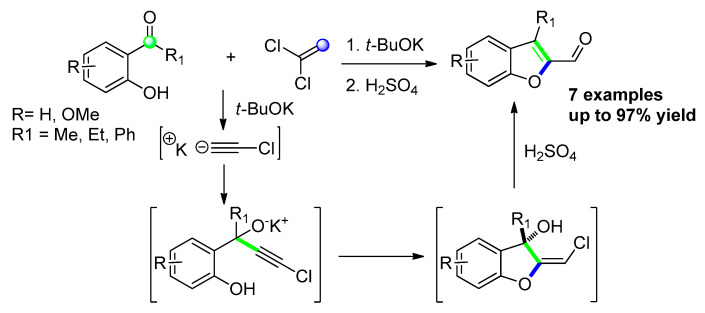
Synthesis of benzofurans via addition of 1,1-dichloroethylene to *o*-hydroxyphenones.

The transition metal-free preparation of highly functionalized benzofurans by a unique and connective transformation has been reported. Base-catalyzed condensation of *o*-hydroxyphenones with 1,1-dichloroethylene generated the corresponding chloromethide benzofurans. These labile intermediates underwent a facile rearrangement into benzofuran carbaldehydes, under mild acidic conditions [124] (Scheme 37).

**Scheme 38:**
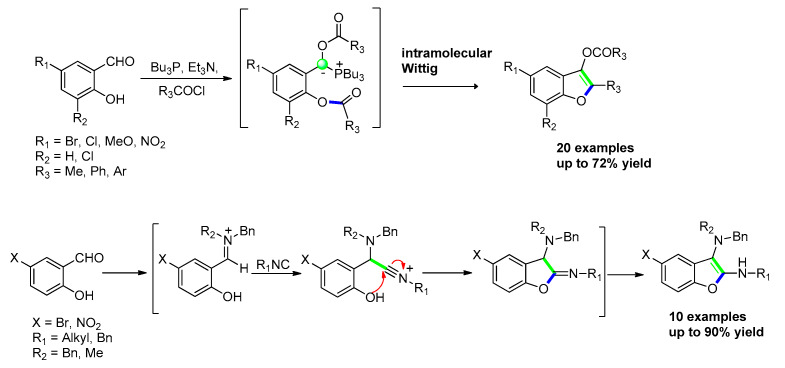
Synthesis of benzofurans via reactions with salicyldehydes.

The preparation of new types of highly functional benzofurans was realized via intramolecular Wittig reactions with the corresponding ester functionality. The key phosphorus ylide intermediate presumably resulted from the addition of Bu_3_P toward salicylaldehydes followed by acylation and deprotonation. The umpolung reactivity of carbonyl carbon of the aldehyde allowed the synthesis of functional benzofurans [125] (Scheme 38).

In an alternative approach, the addition of an isocyanide on an iminium ion intermediate, formed from an electronpoor salicylaldehyde derivative and a secondary amine in the presence of silica gel, proceeded smoothly at room temperature and afforded benzofuran derivatives in high yields [126] (Scheme 38).

#### 3.2.4. Transition-Metal-Catalyzed Approaches: [Rh(II)] Catalyzed Addition of *N*-Sulfonyl-1,2,3-Triazole

**Scheme 39:**
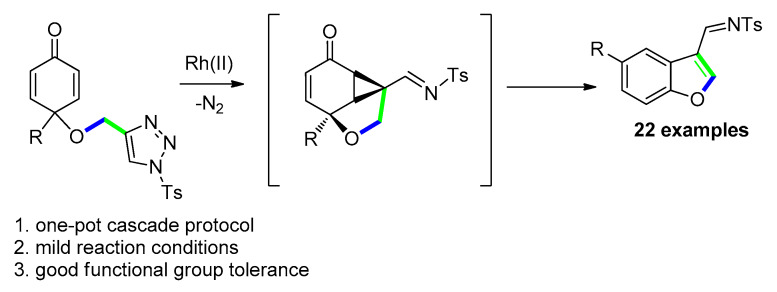
Synthesis of benzofurans via [Rh]-catalyzed addition of *N*-sulfonyl-1,2,3-triazole.

A rhodium-catalyzed intramolecular denitrogenative transannulation of *N*-sulfonyl-1,2,3-triazole-tethered cyclohexadienones has been described for the synthesis of benzofurans and cyclopropa[cd]indole-carbaldehydes in an operationally simple procedure. Remarkably, the reaction pathway is fully dependent on heteroatom (O or N) in the linker between the cyclohexadienone unit and triazole moiety. In the case of *O*-linked triazoles, a cascade sequence consisting of intramolecular cyclopropanation and rearrangement took place, leading to the formation of benzofurans [127] (Scheme 39). 

#### 3.2.5. Transition–Metal Catalyzed Approaches: [Cu(I)]-Catalyzed Addition to *o*-Hydroxybenzophenones/Salicylaldehydes

**Scheme 40:**
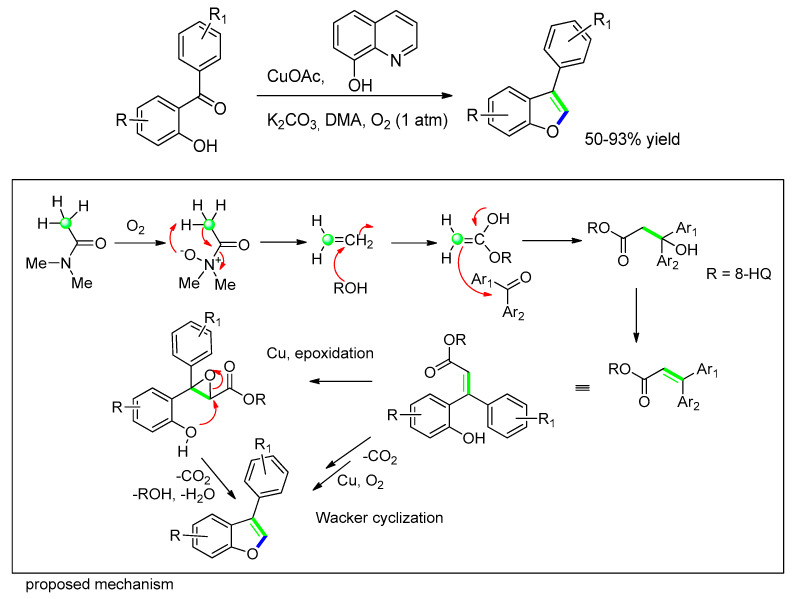
[Cu(I)]-catalyzed synthesis of benzofurans via addition of ketene to *o*-hydroxybenzophenone.

Dominguez and co-workers have described the synthesis of a series of 3-arylbenzofurans [128], using *o*-hydroxy-benzophenone, CuOAc (50%mol), 8-HQ (8-hydroxyquinoline) (50%mol), and K_2_CO_3_ (1 equiv), in DMA (*N*,*N*-dimethoxyacetamide), at 140 °C, under O_2_ atmosphere. The optimized conditions were extended to different diarylketone derivatives affording benzofurans in good yields, in which halogen, alkyl, and alkoxy functional groups were well tolerated under these oxidative conditions. It was demonstrated that DMA took part to reaction furnishing the additional carbon which was involved through ketene intermediate to the formation of 2-hydroxy-α-phenylstyrene or ester α,β-unsaturated. In the last step, the Cu-catalyzed oxidation of the double bond or Cu-catalyzed Wacker cyclization gave rise to benzofurans (Scheme 40). 

As it was said before, the catalytic functionalization of unactivated C-H bonds is an increasingly viable method for organic synthesis. In particular, C-H activations that lead to the formation of C–O bonds have recently provided step-economical access to substituted phenols. A versatile ruthenium(II) complex, [{RuCl_2_(p-cymene)}_2_], in presence of PhI(OTFA)_2_ as the terminal oxidant in DME allowed the preparation of different salicylaldehydes by a site selective C-H oxygenations with weakly-coordinating aldehydes. The challenging C–H functionalizations proceeded with high chemoselectivity by rate-determining C–H metalation.

**Scheme 41:**
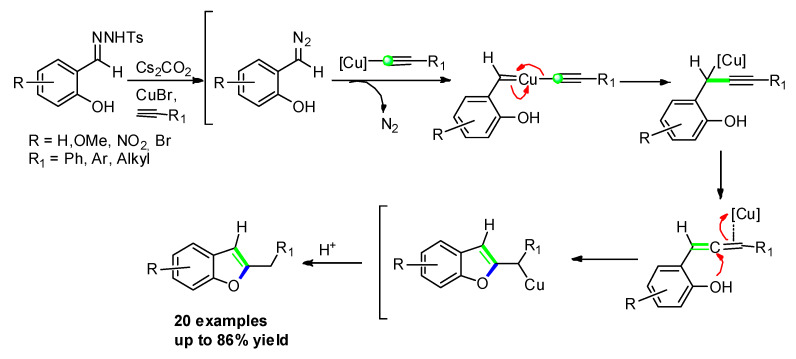
[Cu(I)]-catalyzed synthesis of benzofurans via addition of acetylene to *N*-tosylhydrazones.

The new method featured an ample substrate scope, which set the stage for the step-economical preparation of various heterocycles, among these benzofurans [129]. Wang J. and coworkers [130] developed a new method to prepare benzofurans by using an economically convenient ligand-free CuBr which catalyzed coupling/cyclization of terminal alkynes with *N*-tosyl-hydrazones, derived from *o*-hydroxybenzaldehydes (Scheme 41). *N*-tosylhydrazones were involved in the synthesis of substituted allenes via Cu(I)-catalyzed coupling of with terminal alkynes [131]. A wide range of functional groups on the aryls and alkynes was found to tolerate the reaction conditions. 

**Scheme 42:**
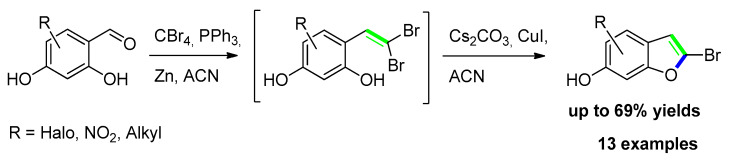
[Cu (I)] catalyzed synthesis of benzofurans via Ramirez olefinations.

Previously, we reported the synthesis of substituted 2-bromobenzofuran compounds from the intramolecular cyclization of gem-dibromoalkenes (prepared via Ramirez olefination) and the subsequent Suzuki cross-coupling for the synthesis of poly-substituted benzofurans (see Section 2.2.3). The aforementioned methods require additional protection–deprotection techniques and are less divergent. An overcoming advanced procedure was envisioned by Lee and co-workers proposing a divergent-pooled route for benzofuran analogues, using 2-bromo-6-hydroxybenzofurans, which were prepared in a one-pot sequence of reactions, using a modified Ramirez olefination and the intramolecular cyclization of the derived *gem*-dibromoalkenes. The best results were obtained when using Cs_2_CO_3_ (3.5 equiv.) and CuI (5 mol %) at 85 °C, giving the desired cyclized compounds with complete selectivity in 65% yield [132] (Scheme 42). 

#### 3.2.6. Miscellaneous

**Scheme 43:**
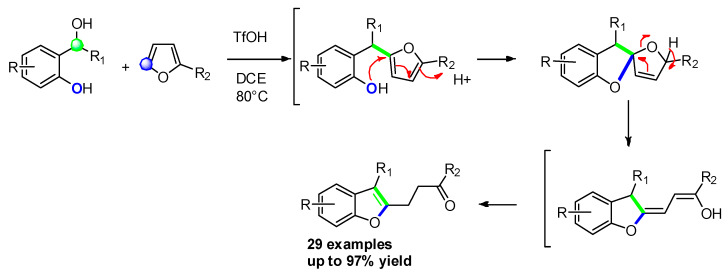
Acid-catalyzed synthesis of benzofurans via salicyl alcohols and furans.

The Brönsted acid-catalyzed cascade synthesis of densely substituted benzofurans from easily available salicyl alcohols and biomass-derived furans has been performed. The disclosed sequence included the formation of 2-(2-hydroxybenzyl)furans that quickly rearranged into functionalized benzofurans. The established protocol was applied for the total synthesis of sugikurojinol B [133] (Scheme 43).

**Scheme 44:**
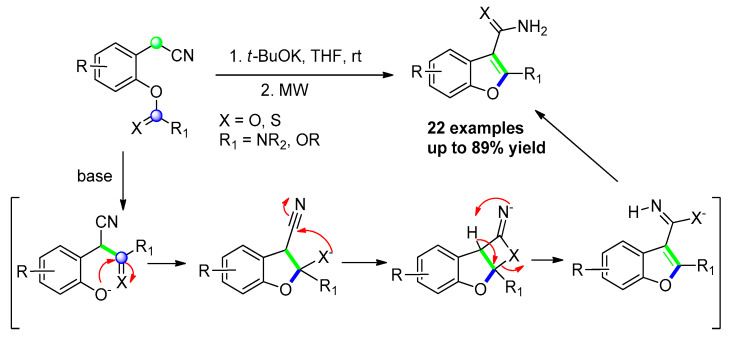
Synthesis of benzofurans via *o*-(cyanomethyl)phenol derivatives.

Chi and co-workers, in 2012, described a convenient method of synthesizing C2-substituted benzofurans from carbamate of 2-hydroxyphenylacetonitrile. In situ two-step reactions using *t*-BuOK in the absence of oxygen and microwave/silica gel treatment provided several C2-derivatized benzofurans in 52–89% yields. Furthermore, straightforward purification of final product by filtration from silica gel avoided the need for column chromatography. This method is quite convenient, because various starting compounds could be easily prepared from commercially available carbonyl chlorides, such as carbamoyl chloride, thiocarbamoyl chloride, chloroformate, and acid chloride, and because further derivatization of benzofurans at the C3 position could be used to find biologically active benzofurans [134] (Scheme 44). A similar mechanism was reported in a procedure of a Pd-catalyzed three-component coupling reaction of *o*-(cyanomethyl)phenol, aryl halide, and carbon monoxide [135].

### 3.3. O–C2 and C3–C3a Bond Formation: (Route b + d)

**Scheme 45:**
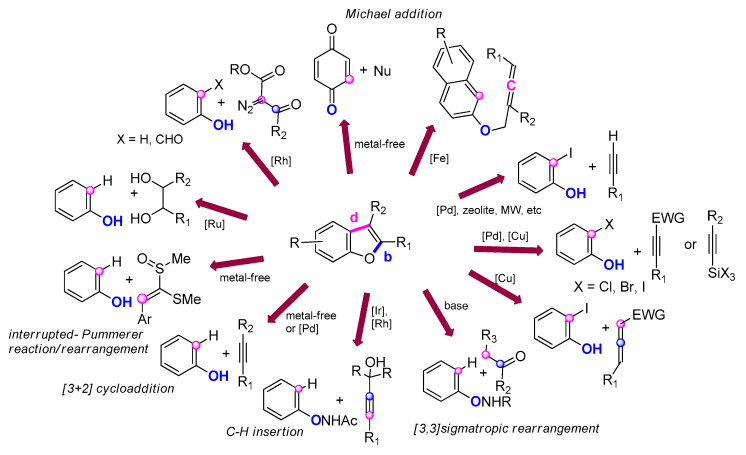
Approaches for O–C2 and C3–C3a bond formation.

The main approaches for the formation of O–C2 and C3–C3a bond dealt in this section are summarized below (Scheme 45).

#### 3.3.1. From *o*-Halophenols and Terminal Alkynes

Pd-catalyzed one-pot synthesis from 2-halophenols and terminal alkynes by a Sonogashira coupling cyclization sequence is a useful and reliable way to construct 2-substituted benzo[b]furans [136,137]. Furthermore, 2-Iodo- and 2-bromophenols have been widely used as 2-halophenols.

**Scheme 46:**
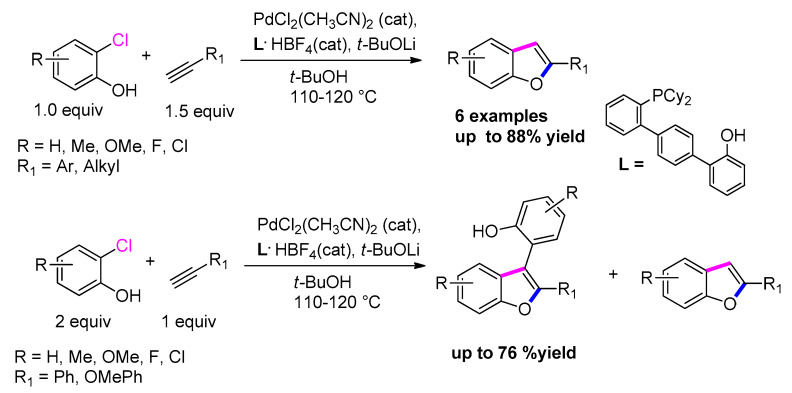
Synthesis of benzofurans via o-halophenol and terminal alkynes.

A catalyst composed of Pd and hydroxyterphenylphosphine was found to be effective for one-pot benzo[b]-furan synthesis from 2-chlorophenols and alkynes [138]. Moreover, 2,3-Disubstituted benzofurans possessing 2-hydroxyphenyl moiety at the C-3 position were synthesized from readily available 2-chlorophenols and terminal alkynes by hydroxy-directed *o*-Sonogashira coupling and subsequent oxypalladation/reductive elimination, using Pd-dihydroxyterphenylphosphine as the catalyst. The catalyst accelerated not only the Sonogashira coupling but also the introduction of 2-hydroxyphenyl group at the C-3 position of benzofuran [139] (Scheme 46).

**Scheme 47:**
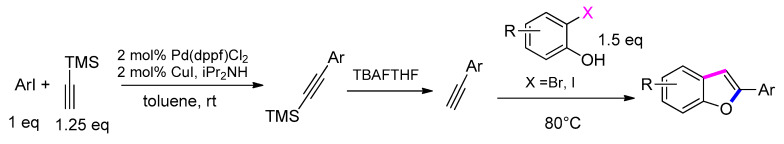
Synthesis of benzofurans via multicatalytic system of o-halophenol and terminal alkynes.

The development of a multicatalytic one-pot synthesis of 2-arylbenzofurans starting from aryl halides and 2-halophenols (bromide or frequently iodide) has been described. The protocol involved two Sonogashira coupling reactions, followed by 2-ethynylphenol cyclization, leading to 2-arylbenzofuran derivatives (Scheme 47). The process occured smoothly under mild conditions, giving products in good yields, and was applied to many 2-arylbenzofurans substituted both at 2-aryl position and in the benzodifuran moiety. Substituents such as halogens, hydroxyl, cyano, nitro, and amino groups were tolerated, enabling further functionalization of the system [140,141].

A multicatalytic system was also used in a cascade transformation of polyenynes into a polyaromatic structure [142]. Nanoparticles of Pd doped by carbon [143] or supported by *N*,*O*-dual-doped hierarchical porous carbon [144], as well as NpPd in water copper- and ligand-free [145], have all been new catalytic systems used to generate benzofurans. 

In 2013, Larock proposed a one-pot three component MW assisted protocol to generate 2,3-disubstituted benzofurans [146]. MWs were used by Elofsson to assist the synthesis of benzofuran core of some natural products [147]. Furthermore, 2-TMS-benzofuran was used in the asymmetric synthesis of the natural product (+)(R)-concentricolide [148]. Moreover, syntheses of benzofurans were proposed in good yield and tolerance of functional groups, using CuI, diaminecyclohexane, and KO*t*Bu, in 1,4-dioxane condition [149] or Cu scorpionate complex and P450 mediated oxidation [150], to generate a methylene-bridged bis-benzofuran system. 

#### 3.3.2. From *o*-Halophenols and Internal Alkynes

**Scheme 48:**
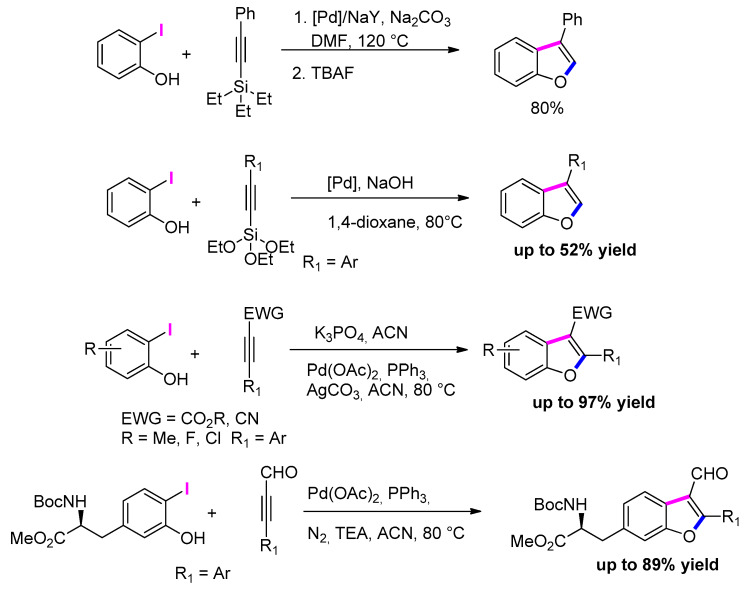
Synthesis of benzofurans via *o*-halophenol and internal alkynes.

Among all methods reported to obtain selectively 2,3-subsituted benzofurans, the Larock procedure, starting from 2-iodophenols and internal alkynes, appeared the most versatile procedure. However, these procedures rely on the use of soluble palladium catalysts; thus, they involve significant difficulties, including the high contamination of the products by palladium and ligand, which is not tolerable in the context of biological applications. Obviously, an analogous catalytic heterogeneous method would eliminate all of these drawbacks. The easily homemade [Pd(NH_3_)_4_]/NaY catalyst appeared to be the best choice for both indoles and benzofurans syntheses, even in reactions where the original Larock procedure failed and for which previous successes required the use of expensive ligand systems [151] (Scheme 48). Recently, Ghosh’s group described a convenient one-pot tandem procedure, a Hiyama alkynylation/cyclization reaction of 2-iodophenol with a range of triethoxysilylalkyne compounds in the presence of palladium acyclic diaminocarbene triflate complexes, which produced 2-substituted benzofurans [152]. A novel approach was developed for the synthesis of 2-substituted-3-functionalized benzofurans, in which the first step was the conjugate addition of phenol to an ynone in the presence of a base (K_3_PO_4_ gave the highest yield). Subsequently, an intramolecular Heck reaction (Pd(OAc)_2_, PPh_3_, Ag_2_CO_3_ in ACN) gave rise to the benzofuran core in a good high yield (up to 97%). This strategy was further applied in the first enantioselective total synthesis of Daphnodorin B [153].

Recently, the same approach was used with the iodo-derivative of tyrosine and several propargyl aldehydes. The atmosphere applied to the reaction medium directly influenced the formation of the products. When an inert atmosphere of nitrogen was applied, a 2-aryl-3-formyl-5-alanylbenzofuran core was selectively obtained via a Heck intramolecular reaction, while under a carbon monoxide atmosphere, the reactions led exclusively to 6-alanyl-2-arylflavone derivatives via reductive intramolecular acylation [154] (Scheme 48).

#### 3.3.3. From *o*-Halophenols and Allenes

**Scheme 49:**
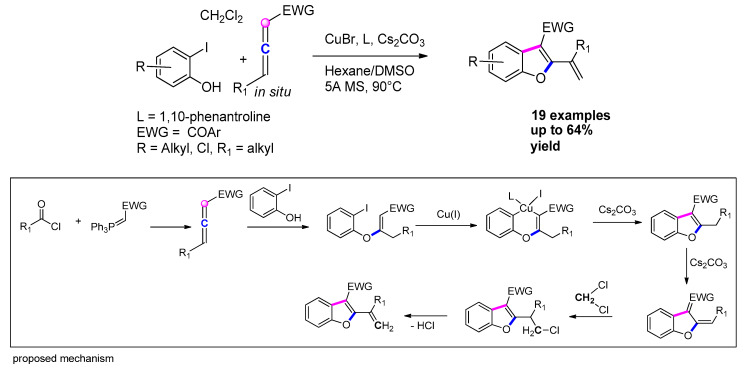
Synthesis of benzofurans via *o*-halophenol and allenes.

Overall, 2-vinylbenzofurans have been synthesized via the copper-catalyzed one-pot, three-component reactions of *o*-iodophenols, in situ generated allenes, and dichloromethane. Cascade transformation of oxa-Michael addition, C-arylation, and sp^3^ C−H/sp^3^ C−Cl conversion-based vinylation has been involved in realizing the construction of this 2-vinylbenzofuran framework [155] (Scheme 49).

#### 3.3.4. From Phenols: *O*-aryloxime/[3,3]-Sigmatropic Rearrangement/Cyclization 

**Scheme 50:**
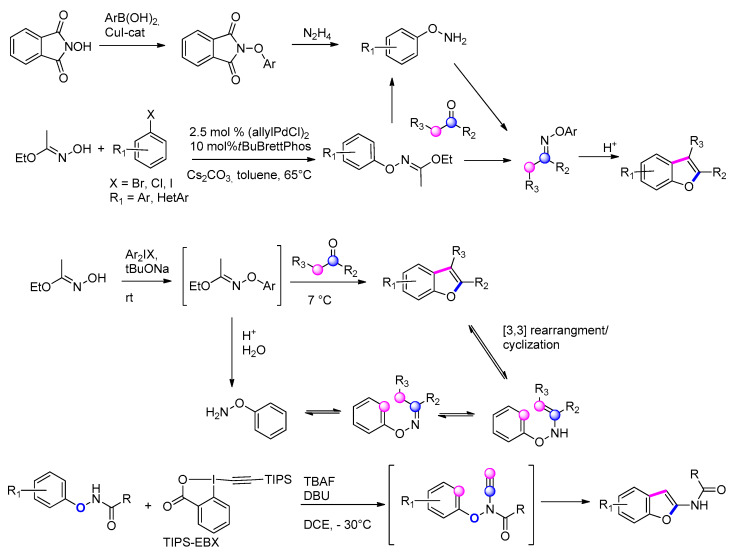
Synthesis of benzofurans via oxime formation/[3,3]-sigmatropic rearrangement/cyclization.

One century after their discovery, [3,3]-sigmatropic rearrangements occupy an irreplaceable role in the synthesis of complex organic molecules and continue to be intensively investigated. Among the methods available to prepare benzofurans one of the most synthetically accessible involves [3,3]-sigmatropic rearrangement of preformed *O*-aryl oxime ethers promoted by Brønsted or Lewis acids [156]. Although high efficiency has been achieved in the synthesis of indoles, as well as benzofurans, under mild reaction conditions via cleavage of O−N bonds, a unified approach to access diverse oxa-heterocycles is highly desirable. The introduction of an O−N bond may encompass the elevated temperature required by the classical Claisen [3,3]-sigmatropic rearrangement. The other challenge is that the annulation/aromatization may not occur readily after the rearrangement step. *O*-aryl oxime ethers was synthesized by the Cu-catalyzed arylation of *N*-hydroxyphthalimide with arylboronic acids, followed by cleavage with hydrazine [157] (Scheme 50). Buchwald and co-workers recently reported a more general palladium-catalyzed arylation of ethyl acetohydroxamate with aryl halides in the presence of air-sensitive alkyl-arylphosphine ligands. Ethyl acetohydroxamate served as an efficient hydroxylamine equivalent for C−O cross-coupling, thereby allowing for the preparation of O-arylhydroxylamines from simple aryl halides. Short reaction times and broad substrate scope, including heteroaryl coupling partners, allowed access to *O*-arylhydroxylamines that would be difficult to prepare in a single step by traditional methods. Moreover, the *O*-arylated products so formed could be directly transformed into substituted benzofurans in a single operation [158]. Ethyl acetohydroxamate was efficiently arylated with diaryliodonium salts at room temperature under transition-metal-free conditions. The obtained *O*-arylated products were reacted in situ with ketones, under acidic conditions, to yield substituted benzo[b]furans through oxime formation, [3,3]-rearrangement, and cyclization, in a fast and operationally simple one-pot fashion, without using an excess of reagents. Alternatively, the *O*-arylated products could be isolated or transformed in situ to aryloxyamines or *O*-arylaldoximes. The methodology was applied to the synthesis of Stemofuran A and the formal syntheses of Coumestan, Eupomatenoid 6, and (+)-machaeriol B [159]. 

An efficient method to selectively construct benzofuran and dihydrobenzofuro[2,3-d]oxazole derivatives has been successfully established by means of base-controlled cyclization of *N*-phenoxyamides with 1-[(triisopropylsilyl)ethynyl]-1,2-benziodoxol-3(1*H*)-one (TIPS-EBX). *N*-phenoxyamides, as multitasking reagents, have triggered two different cascade-reaction sequences. This was the first example of using TIPS-EBX for the transformation of C(sp) to either C(sp^2^) or C(sp^3^) under metal-free conditions [160,161].

#### 3.3.5. Via Transition-Metal-Catalyzed Annulation of *N*-Aryloxyacetamides and Propargyl Alcohols

Propargylic alcohols are some of the most useful building blocks with two functional groups. These units have been involved in numerous cascade synthetic transformations in organic chemistry, providing an opportunity to discover novel cascade processes [162].

**Scheme 51:**
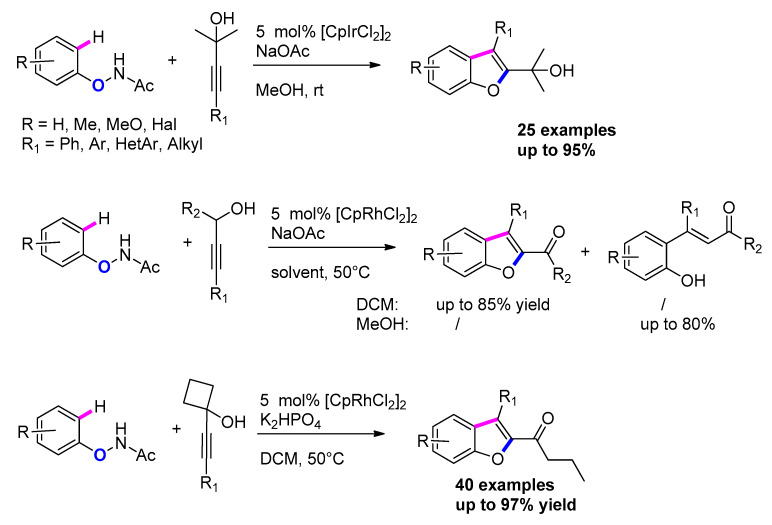
Synthesis of benzofurans via TM-catalyzed annulation of *N*-aryloxyacetamides and propargyl alcohols.

In 2018, Yi revealed an efficient and mild Ir(III)-catalyzed C−H annulation of *N*-aryloxyacetamides with tertiary propargyl alcohols to deliver benzofurans [163], in which the efficiency of protocol was influenced by the position and the nature of substituents on phenol. In the same year, Yi developed the Rh(III)-catalyzed and solvent-controlled C−H functionalization of *N*-aryloxyacetamides with secondary or primary propargyl alcohols for the divergent synthesis of chalcones and benzofurans [164]. By virtue of a synergically dual-directing-group (the O−NHAc part and the hydroxyl group)-assisted strategy, the efficient and practical Rh(III)-catalyzed regioselective redox neutral C−H functionalization of diverse *N*-phenoxyacetamides with propargyl alcohols has been realized, which led to the divergent synthesis of privileged benzofuran and chalcone frameworks in a solvent-controlled chemoselective manner (Scheme 51). Experimental and computational studies revealed that the formation of the hydrogen bonding between dual directing groups and the subsequent coordination interaction between the hydroxyl group and the Rh(III) catalyst play a decisive role in promoting the regioselective migratory insertion of the alkyne moiety. Thereafter, two solvent-controlled switchable reaction pathways occured to deliver the corresponding products with excellent chemoselectivity.

A cascade [3 + 2] annulation of *N*-aryloxyacetamides with 1-alkynylcyclobutanols via Rh-(III)-catalyzed redox-neutral C−H/C−C activations, using internal oxidative O−NHAc and −OH as the dual directing groups, has been achieved, as well, with the subsequent ring-opening of cyclobutanol. This reaction, performed with [Rh]-complex and KH_2_PO_4_ in DCM, provided an efficient and regioselective approach to benzofuran derivatives, with good functional group compatibility and high yields [165].

#### 3.3.6. Metal-Free [3 + 2] Annulation of Phenols with Acetylenes

The metal-free [3 + 2] annulation of phenols with propargylic alcohols generated benzofurans as well as naphthofurans, in a highly atom-economy manner. This reaction utilized C1 and C2 carbons of propargylic alcohols for the annulation in a two-step process involving (i) an acid catalyzed intermolecular C–C bond formation between C1 of the propargylic alcohol and the α-position of phenol or β-naphthol, i.e., α-propargylation; and (ii) a base-catalyzed intramolecular O–C bond formation between C2 of the propargylic alcohol and –OH of phenol or naphthol [166].

**Scheme 52:**
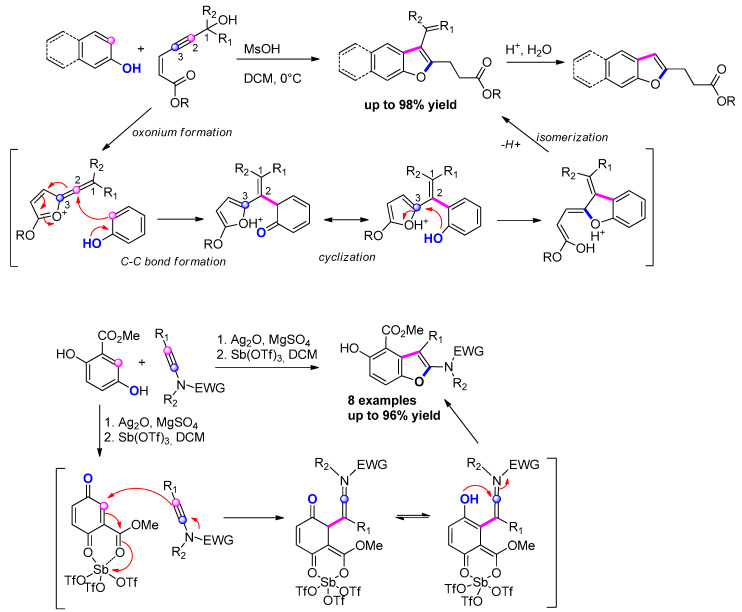
Synthesis of benzofurans via metal-free [3 + 2] annulation of phenols and acetylenes.

An unprecedented acid-mediated cascade [3 + 2] annulation process for the generation of complex naphthofurans (benzofurans) from propargylic alcohols and β-naphthols (resorcinols) was proposed by Baire and co-worker [167] (Scheme 52). They described the synthesis of naphthofurans (and benzofurans) in a cascade manner, via an annulation utilizing C2 and C3 carbons of propargylic alcohols with β-naphthols (resorcinols).

Densely substituted amino-functionalized benzofurans were concisely accessed via the first one-pot domino oxidation/[3 + 2] cyclization of a hydroquinone ester and easily accessible ynamides under mild conditions in short time. The complex benzofurans were able to be efficiently synthesized, all from simple and inexpensive starting materials, in two steps [168].

#### 3.3.7. [Pd]-Catalyzed [3 + 2] Annulation of Phenols with Internal Alkynes

While the transition metal (TM)-catalyzed annulation between aniline and unactivated alkynes provides indoles andtheir derivatives easily, a detailed survey of the literature reveals that the corresponding one step synthesis of benzofurans from readily available phenols and unactivated alkynes has remained elusive so far. The reactivity of phenol toward unactivated alkynes presents challenges as the participation of an unfavorable four-membered oxygen-containing metallacycle, the difficulties associated with the formation of the C-O bond through reductive elimination of the putative Pd(II) intermediates, and the sensitivity of phenols to strong oxidants like TMs.

Despite these cumulative challenges, Sahoo and co-workers [38,169] developed an unprecedented one-step synthesis of benzofurans by the Pd-catalyzed oxidative annulation of readily accessible phenols and unactivated internal alkynes.

**Scheme 53:**
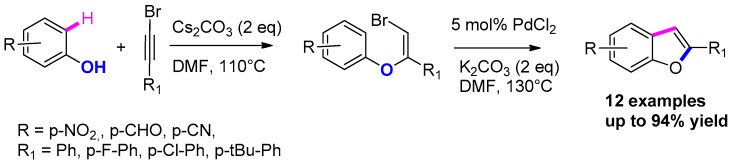
Synthesis of benzofurans via [Pd] catalyzed [3 + 2] annulation of phenols and acetylenes.

Moreover, benzo furans were prepared in one-pot, based on the addition/palladium-catalyzed C-H bond functionalization of phenols with bromoalkynes. The addition reactions of phenols to bromoalkynes generated (*Z*)-2-bromovinyl phenyl ethers in high yields with excellent regio- and stereoselectivity. The obtained (*Z*)-2-bromovinyl phenyl ethers subsequently proceeded by cyclization, affording 2-substituted benzofurans in good yields. It is important to note that the transformation of phenols with bromoalkynes into benzofurans could be carried out in one-pot with a simple and efficient tandem procedure [170] (Scheme 53).

Palladium-catalyzed oxidative annulations between phenols and alkenylcarboxylic acids produced a library of benzofuran compounds. Depending on the nature of the substitution of the phenol precursor, either 2,3-dialkylbenzofurans or 2-alkyl-3-methylene-2,3-dihydrobenzofurans were synthesized with excellent regioselectivity [38,171,172,173].

#### 3.3.8. Via Interrupted Pummerer Reaction/[3,3] Sigmatropic Rearrangement/Cyclization

The Pummerer reaction is a reaction of an alkyl sulfoxide with a Lewis acidic activator (LA+), such as acid anhydride, to yield a α-functionalized alkyl sulfide. Interrupted Pummerer reactions are different from other Pummerer-type reactions in terms of the reaction mode: The cationic sulfur center is directly attacked, or interrupted, by a nucleophile [174]. In 2010, Yorimitsu and co-workers prepared 2-methylthio-3-trifluoromethyl-substituted benzofurans from phenol and ketene dithioacetal monoxides (KDM) [175]. Subsequently, the same group extended the methodology by using a wide range of KDMs activated by trifluoroacetic anhydride (TFFA), in order to avoid the fast decomposition of dicationic intermediate [176,177,178].

**Scheme 54:**
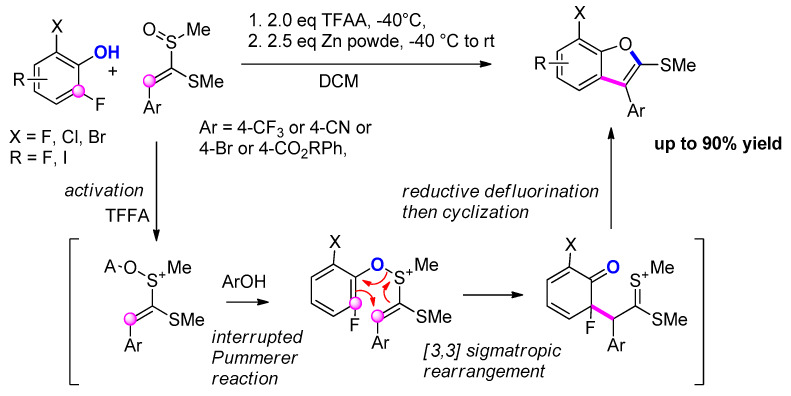
Synthesis of benzofurans via interrupted Pummerer reaction/[3,3]-sigmatropic rearrangement/annulation of perfluorophenols.

In 2018, they accomplished a facile synthesis of fluorinated benzofurans from polyfluorophenols by means of a sigmatropic dearomatization/defluorination strategy composed of three processes: (1) interrupted Pummerer reaction of ketene dithioacetal monoxides, activated by TFFA, with polyfluorophenols followed by [3,3] sigmatropic rearrangement; (2) Zn-mediated smooth reductive removal of fluoride from the dearomatized intermediate; and (3) acid-promoted cyclization/aromatization to lead to benzofuran in 90% overall yield. Some of the fluorinated benzofurans were transformed by utilizing the 2-methylsulfanyl moieties [179] (Scheme 54).

**Scheme 55:**
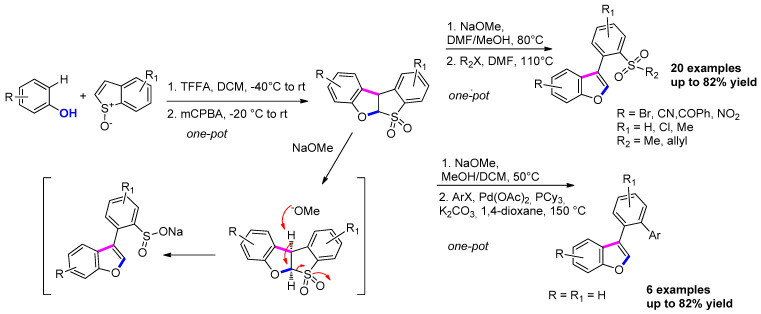
Synthesis of benzofurans via interrupted Pummerer reaction/[3,3]-sigmatropic rearrangement/annulation/desulfinative reactions.

Procter’s group reported a transition-metal-free synthesis of benzofurans from benzothiophenes and phenols which exploited the unique reactivity of sulfoxides [180]. Through a sequence involving an interrupted Pummerer reaction and [3,3] sigmatropic rearrangement, phenols were combined with readily accessible, yet synthetically unexplored, benzothiophene *S*-oxides to provide 3-arylated benzofurans. The products from this approach underwent subsequent functionalization, to gain access to a range of important benzofuran derivatives (Scheme 55). Sulfinate salts are a class of versatile compounds that have recently found application as coupling partners in palladium-catalyzed cross-coupling reactions. In fact, they were subjected to the subsequent desulfinative cross-coupling of substituted aryl halides, known to be easily available [181]. This approach established sulfoxides as a traceless activating group for C−H functionalization in this method. Thus, the intermediate aryl sulfinates, formed from treatment of the sulfones with base, underwent desulfinative palladium-catalyzed cross-coupling in the same pot, to provide the desired biphenyl benzofurans. This procedure gave good-to-excellent yields for all substrates tested; *o*-, *m*-, and *p*-substituted substrates all gave similarly high yields. It is worth noting that no trace of the sulfoxide group was present in the unreacted starting material.

#### 3.3.9. Via Fries-type O-C Rearrangement/Michael Addition of Phenols

Recently, the direct synthesis of naphthofurans and benzofurans was reported from readily available phenols and *α*-haloketones. It was promoted by titanium tetrachloride (TiCl_4_) which combined Friedel–Crafts-like alkylation and intramolecular cyclodehydration into one step. High levels of regioselectivity, broad substrate scope, and moderate-to-excellent yields were obtained [182].

**Scheme 56:**
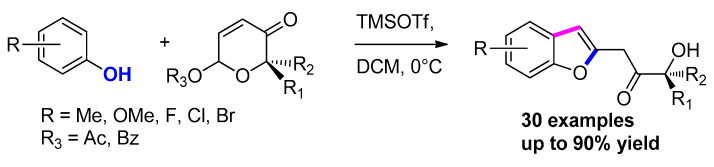

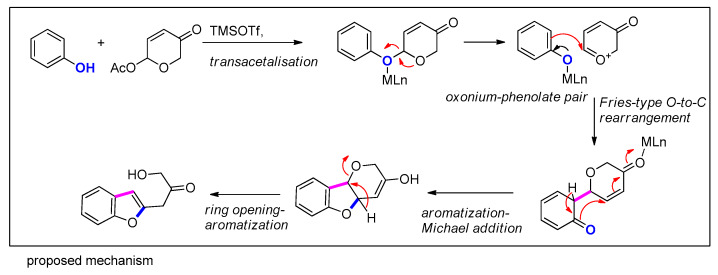
Synthesis of benzofurans via Fries-type O-C rearrangement/Michael addition.

An unusual and facile approach for the synthesis of 2-benzofuranyl- 3-hydroxyacetones from 6-acetoxy-β-pyrones and phenols was described by Ramasastry [183] (Scheme 56). The synthetic sequence involved a cascade transacetalisation, Fries-type O–C rearrangement followed by Michael addition, and ring-opening aromatization. The unexpected cascade event also provided new possible considerations in the β-pyrone-involved organic synthesis.

Seggi and co-workers reported that 3-(2-bromoethyl)benzofurans were readily obtained from commercially available bis[(trimethylsilyl)oxy]cyclobutene and various phenols via a Brønsted acid-mediated nucleophilic addition−carbocyclic rearrangement cascade reaction; this is a one-pot, metal-free process that operates in mild conditions [184]. In the presence of a Brønsted acid, 2-hydroxycyclobutanone and its precursor bis[(trimethylsilyl)oxy]cyclobutene behaved as electrophilic acceptors for intermolecular nucleophilic addition, followed by a ring closure−ring fission process. This mild and facile strategy was applied for the synthesis of a series of 5-HT serotonin receptor agonists, underlining its potential for the syntheses of bioactive compounds and natural products.

#### 3.3.10. Via [Ru]-Catalyzed C–H Alkylation of Phenols with 1,2-Diols

**Scheme 57:**
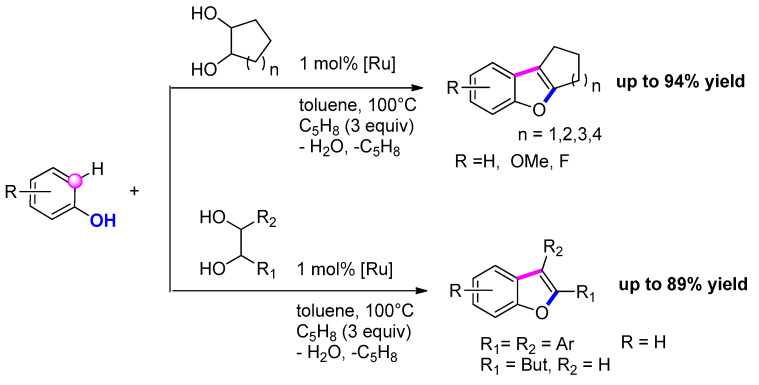

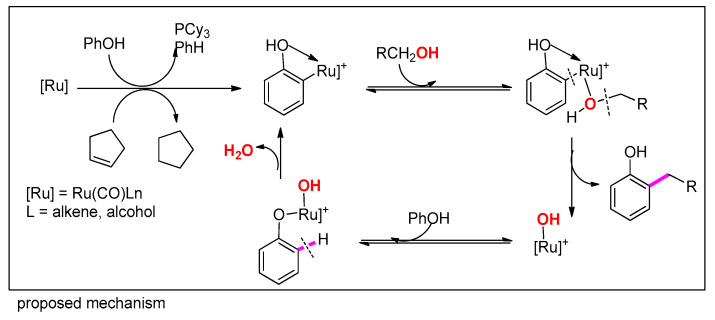
Synthesis of benzofurans via [Ru]-catalyzed C-H alkylation of phenols with 1,2-diols.

Alcohols have been rarely employed as the substrate for the catalytic C−H coupling reactions, because of their tendency for undergoing energetically more favorable alkoxylation and oxidation reactions over the respective C−O bond cleavage reaction. Yi and co-workers discovered an exceptionally selective dehydrative C−H alkylation reaction of alkenes with alcohols that was catalyzed by a well-defined cationic ruthenium hydride complex [(C_6_H_6_)(PCy_3_)(CO)RuH]^+^BF_4_^−^. This cationic Ru−H complex also catalyzed the dehydrative C−H alkylation reaction of phenols with alcohols to form ortho-substituted phenol products so that benzofuran derivatives were efficiently synthesized from the dehydrative C−H alkenylation and annulation reaction of phenols with 1,2-diols [185]. The catalytic C−H coupling method employed cheaply available phenols and alcohols, exhibited a broad substrate scope, tolerated carbonyl and amine functional groups, and formed water as the only byproduct (Scheme 57).

#### 3.3.11. Via [Rh]-Catalyzed Carbene Insertion with Phenols/Salicylaldehydes

**Scheme 58:**
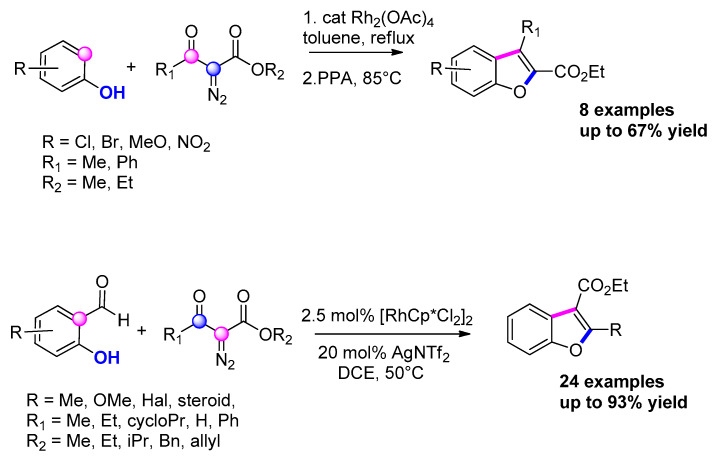

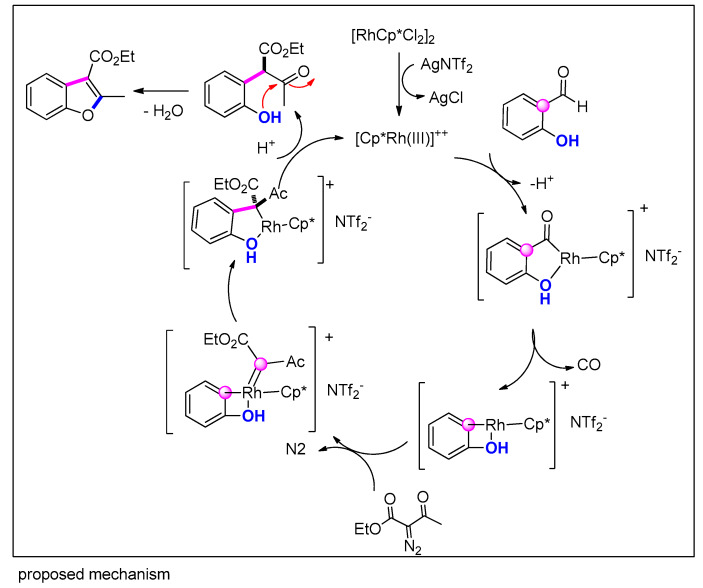
Synthesis of benzofurans via [Ru]-catalyzed carbene insertion with phenols/salicylaldehydes.

Transition-metal carbene X–H insertion reactions (X=N or O) have been employed in the simple conversion of anilines and phenols into indoles and benzofurans, respectively. Thus, copper(II) catalyzed N–H insertion reactions of α-diazo-β-ketoesters with *N*-methylanilines, followed by treatment with acidic ion exchange resin gave indoles. In a similar manner, dirhodium(II) catalyzed O–H insertion reactions of α-diazo-β-ketoesters with phenols, followed by treatment with polyphosphoric acid (PPA) gave benzofurans [186] (Scheme 58).

A Rh(III)-catalyzed annulation between salicylaldehydes and diazo compounds with controllable chemoselectivity was described by Lin and Yao [187]. AgNTf_2_ favored benzofurans via a tandem C−H activation/decarbonylation/annulation process, while AcOH led to a chromones through a C−H activation/annulation pathway. The reaction exhibited good functional group tolerance and scalability. Moreover, only a single regioisomer of benzofuran was obtained due to the in situ decarbonylation orientation effect. Reactions of salicylaldehyde and its cyclic acetals with diazocarbonyl compounds in the presence of copper and rhodium catalysts have been studied. The reaction pathway and product yields were determined by the nature of the initial reactants and catalyst [188] (Scheme 58).

#### 3.3.12. Via Michael Addition/Cyclization of Nucleophiles on Benzoquinones

**Scheme 59:**
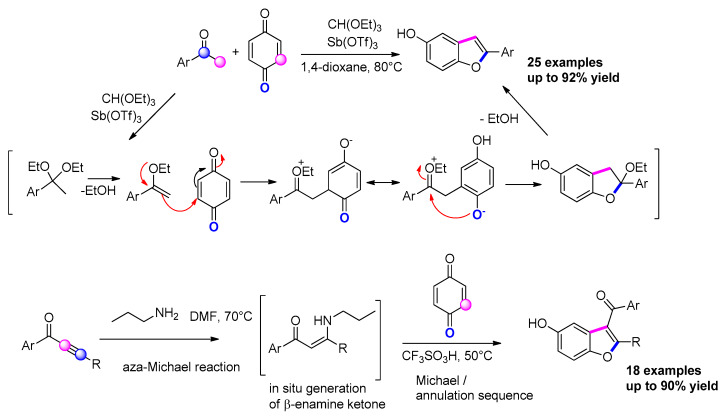
Synthesis of benzofurans via Michael addition/cyclization of nucleophiles on p-benzoquinones.

Benzofuran derivatives were synthesized through the sequential Michael addition and cyclization of 1,3-dicarbonyl compounds with 1,4-benzoquinones. However, ketones are rarely used in this reaction because of their low nucleophilicities. In this study, this problem was solved by utilizing triethyl orthoformate, which enabled the formation of a vinyl ethyl ether as an additive. As a result, the nucleophilicity of ketones increased. Many important 5-hydroxybenzofuran derivatives, not previously available by synthesis, were also prepared by these newly established reactions [189] (Scheme 59).

A convenient metal-free one-pot synthesis of benzofuran derivatives starting from simple ynones has been developed by Cui and co-workers [190]. Various functionalized benzofurans, closely related to bioactive molecules, were obtained in moderate-to-good yields (up to 90%) through aza-Michael/Michael/annulation sequence (a mechanism similar to the previously described method). Preparative scale synthesis of benzofurans was successfully achieved, as well. The application of the benzofuran products was shown by easy transformations to highly functionalized molecules, holding significant promise for medicinal chemistry and organic material chemistry.

**Scheme 60:**
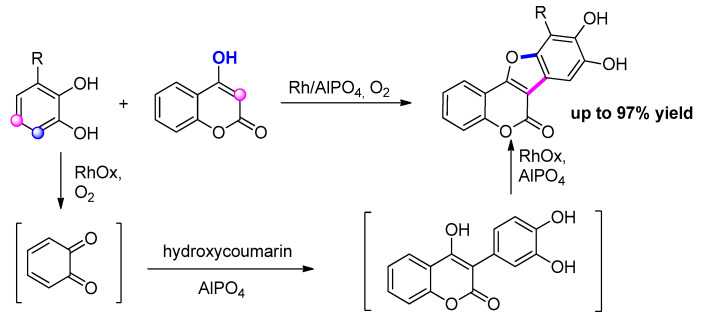
Synthesis of benzofurans via Michael addition/cyclization of nucleophiles on o-benzoquinones.

An efficient synthesis of benzofuran derivatives via the cross-coupling of catechols and hydroxycoumarins in H_2_O, using O_2_ as an ideal oxidant, was reported by Maeno and co-workers (Scheme 60). The above reaction allowed the direct use of substrates without prefunctionalization, involved formation of C–C and C–O bonds in a cascade manner, and afforded H_2_O as the sole by-product. This simple and clean reaction was achieved by the development of an AlPO_4_-supported Rh nanoparticle catalyst. The catalyst was applicable to the synthesis of a wide range of benzofurans. This catalytic method was successfully utilized for total synthesis of flemichapparin C, one of the naturally occurring coumestans exhibiting bioactivity [191].

#### 3.3.13. Via FeCl_3_-Catalyzed Allenic Claisen Rearrangement/ Dehydrogenative Cyclization

**Scheme 61:**
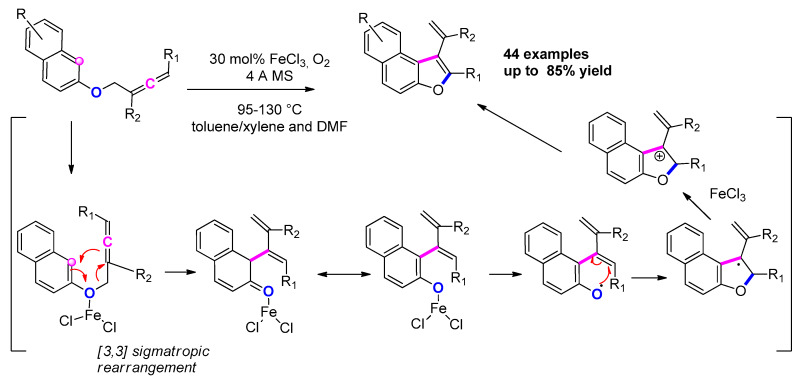
Synthesis of benzofurans via FeCl_3_-catalyzed allenic Claisen rearrangement/ dehydrogenative cyclization.

A FeCl_3_-catalyzed allenic Claisen rearrangement/regio- and chemoselective aerobic dehydrogenative cyclization domino reaction is developed, providing a wide range of 2-aryl/alkyl, 3-(substituted-vinyl)naphtho[2,1-b]-furans in high yields at 95–130 °C in an atom- and step economic fashion. Mechanistic studies suggested that the FeCl_3_ catalyst was responsible for the high regio- and chemoselectivity in reaction. A blue-emitting product showed a quantum yield of 0.95. The reaction proceeded readily on the gram scale, and synthetic applications of the products were also demonstrated [192] (Scheme 61).

## 4. Benzoannulation

This last section reports examples in which the benzofuran scaffold was built through benzannulation or aromatization. Few recent examples were already reported in References [28] and [30].

**Scheme 62:**
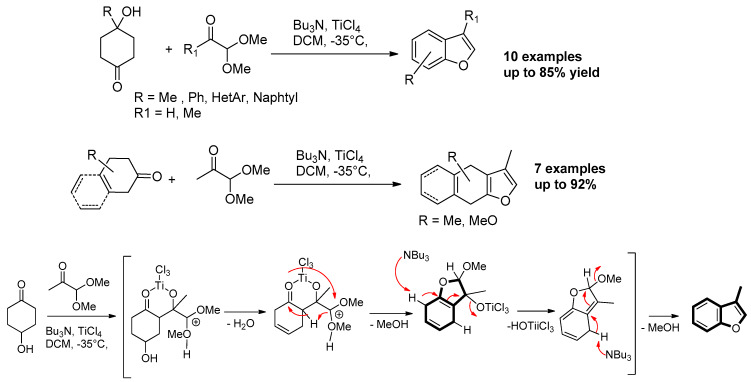
Synthesis of benzofurans via Tanabe-type annulation with cyclohexanone and 1,1-dimethoxy acetone.

Recently, Mehta and co-workers, during an attempt to perform a Tanabe annulation [193] on 4-hydroxy-cyclohexanone with 1,1-dimethoxy acetone, obtained the corresponding 3-methybenzofuran as the only product in 78% yield [194] (Scheme 62). This method was extended to naphthofurans and other related frameworks. The utility of this adaptable methodology was applied to concise syntheses of natural products, stereumene B, paeoveitol D, and (±)-paeoveitol.

**Scheme 63:**
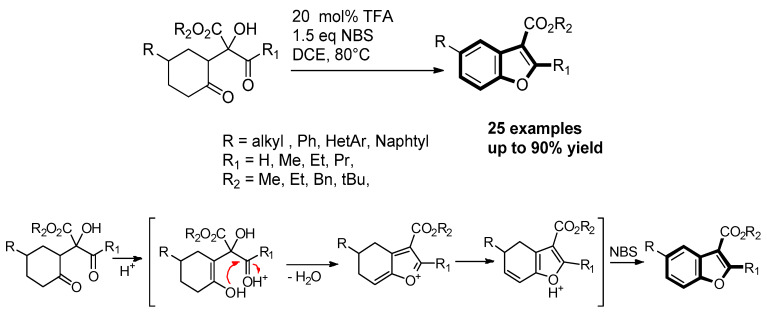
Synthesis of benzofurans via cyclization/oxidative aromatization cascade reaction of 2-hydroxy-1,4-diones.

A novel cascade de novo reaction to access benzofurans from suitable 2-hydroxy-1,4-diones was reported by Sha [195]. The hydroxyl group played a crucial role in the reaction, allowing the dehydration reaction to form the key C-C bond during the cascade reaction process. This facile one-pot method for the preparation of benzofurans in moderate-to-good yields (R_1_ = Me, Ph, 2-naphthyl, etc.; R_2_ = Me, EtO, BnO, etc.; R = H, Et, MeO_2_C, Ph, etc.) runs via cyclization/oxidative aromatization cascade reaction of 2-hydroxy-1,4-diones, using trifluoroacetic acid as a catalyst and *N*-bromosuccinimide as oxidant. Such 2-hydroxy-1,4-diones also showed as a supplement of the Paal–Knorr furan synthesis. A preliminary study was undertaken, as well, to support the proposed mechanism, during which a novel 1,6-conjugate addition reaction was revealed (Scheme 63).

**Scheme 64:**
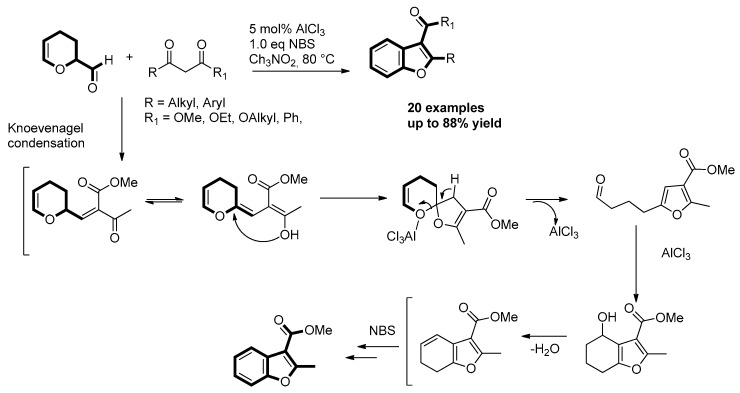
Synthesis of benzofurans via Knoevenagel condensation with acrolein dimer and acetoacetates.

Recently, the coupe Lewis acid/NBS as catalyst and oxidant, respectively, was proposed as partners of reaction for the facile way to construct a six-and-five two-aromatic-ring fused heterocycle, namely benzofuran. Starting from easily available chemicals, acrolein dimer and 1,3-dicarbonyl compounds, 2,3-disubstituted benzofurans were synthesized in good yield (Scheme 64). The method succeded to synthesize two commercial drug molecules, benzbromarone and amiodarone [196].

**Scheme 65:**
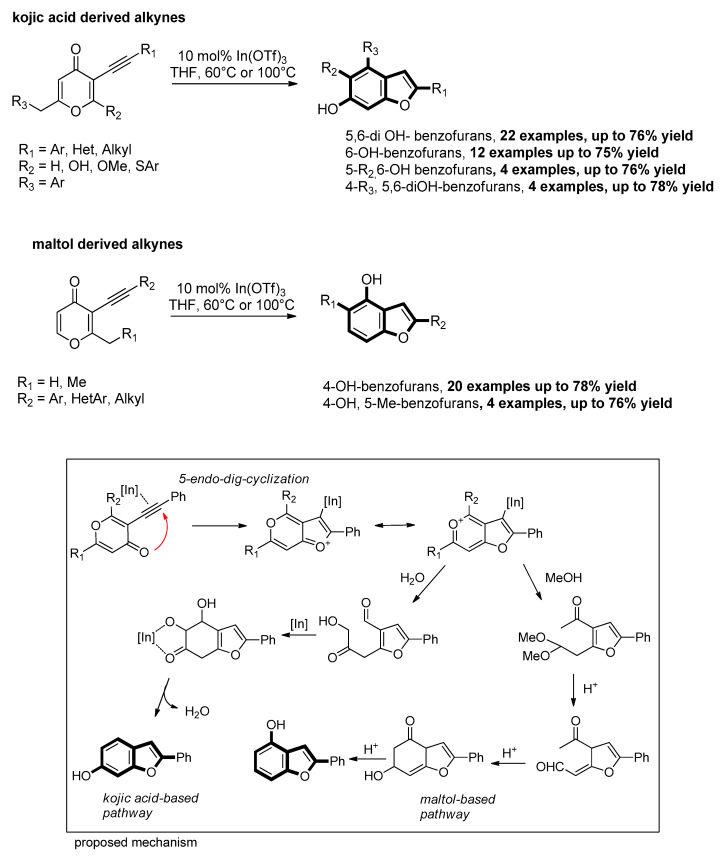
Synthesis of benzofurans via [In]-catalyzed decostructive reorganization strategy.

At last, a very interesting methodology was proposed by Zhu and co-worker in 2020. This is an unprecedented decostructive reorganization strategy for the preparation of hydroxylated benzofurans from either kojic acid or maltol-derived alkynes [197] (Scheme 65). With the aim to develop new dearomatic cascade rearrangement of pyrones, the authors reported a study in which both the benzene and furan rings were simultaneously estabilished via an arene cycloisomerization tandem reaction. A range of substitution patterns was achieved, and a large number of hydroxylated benzofurans were prepared in one-step, with 100% atom economy, enabling a collective total synthesis of different kinds of natural products.

## 5. Conclusions

This review has described recent progress in transition-metal-catalyzed and metal-free couplings for the synthesis of polysubstituted benzo[b]furans. Due to their high efficiency, economy and versatility, transition-metal-catalyzed one-pot processes, especially those involving multiple C–C/C–O bond-forming cascades in an inter-molecular approach, are powerful methods and thus have been extensively investigated. However, the development of more sustainable catalytic systems and more practical synthetic methods, starting from simple and readily available feedstocks, is still highly desirable. Due to the large amount of publications on this topic, a selection of the most relevant had to be done. Hopefully, this review could be a reference of new synthetic strategies which have never appeared in previous reviews.
